# Human live spermatozoa morphology assessment using digital holographic microscopy

**DOI:** 10.1038/s41598-022-08798-6

**Published:** 2022-03-22

**Authors:** Marzena Kamieniczna, Ewa Stachowska, Agata Augustynowicz, Tomasz Woźniak, Maciej K. Kurpisz

**Affiliations:** 1 Institute of Human Genetics, Polish Academy of Sciences, Poznan, Poland; 2 Department of Metrology and Measurement Systems, Institute of Mechanical Technology, Faculty of Mechanical Engineering, Poznan University of Technology, Poznan, Poland

**Keywords:** Biophysics, Cell biology

## Abstract

Digital holographic microscopy (DHM) was applied for the morphological assessment of live intact spermatozoa from fertile and infertile men directly after semen liquefaction. This method allowed us to study the sperm population directly from the sample droplet and not only from the focal plane of the microscope as in classical optical microscopy. The newly implemented 3-dimensional sperm morphological parameters (*head height*, *acrosome/nucleus height, head/midpiece height)* were included in morphological assessment of semen samples from fertile and infertile individuals. The values of the 3D parameters were less variable in fertile men than for infertile ones. DHM was also used to compare the morphological profiles of spermatozoa after applying the “swim-up” and gradient centrifugation techniques. During selection, the most statistically significant differences were observed after separation with a Percoll gradient of 90% and a 60-min “swim-up” procedure versus ‘native’ unfractionated samples. This shows that the developed methodology can be efficiently used for the selection of morphologically sound spermatozoa. The motility type for each spermatozoon was also assessed. The results indicate that the extension of the number of morphological parameters with new 3D parameters and the simultaneous assessment of sperm motility may be valuable addition to sperm examination.

## Introduction

There is a growing interest in investigating human sperm morphology as a way of fertilization success prediction. Sperm morphology data may have high predictive value for male fertility potential since correct sperm morphology dictates the successful outcome of all stages of fertilization (spermatozoa progressive movement, cervical mucus penetration, capacitation, zona pellucida recognition, acrosome reaction, sperm-oocyte fusion, implantation, and the early embryonal stage)^1,2^. Conventional light microscopy shows only a limited degree of correlation between sperm phenotypes and the fertility of an individual^3^. For this reason, new microscopic techniques are being intensively pursued for their utilization in sperm morphology studies^4–7^.

Almost 20 years ago, the MSOME (motile sperm organelle morphology examination) method for studying sperm morphology was introduced. MSOME enables the examination of motile spermatozoa in real time using an inverted light microscope equipped with high-power Nomarski optics enhanced by digital imaging to achieve a magnification level of 6300x^4^. Morphological assessment is conducted with a help of a monitor screen. By means of MSOME, sperm subcellular organelles such as the acrosome, postacrosomal lamina, nucleus, neck, tail and mitochondria can be observed, in most cases qualitatively. This method has been frequently used in ART laboratories to investigate single motile spermatozoa and not the entire fraction of sperm^4^.

The development of optical holography was one of the most remarkable inventions of the XX century. Researchers succeeded in producing holograms in the 1960s, but the method has not found broad application due to its restricted experimental requirements. The advancement of digital holography occurred in the late 1990s when high-speed microprocessors, micro lens mirror arrays and liquid crystal displays became available^5^. Due to its noninvasive, quantitative and label-free nature, quantitative phase imaging has been applied to the measurement of unlabelled cells and tissues^6^. By means of this technique, the features of external structures in live cells can be studied. Digital holographic microscopy (DHM) records and reconstructs the phase and amplitude of the wave front of the laser beam transmitted by the object. The hologram is proportional to the intensity of the interference resulting between the object and reference beams and is acquired by a CCD camera. By the numerical back-propagation of the product between the recorded hologram and a replica of the reference beam, the optical wave front of the object is reconstructed. This allows one to obtain quantitative information about the topographic profile of the object. The presentation of a bovine sperm head for morphometric analysis in quantitative phase-contrast holographic microscopy was reported by Di Caprio^8^ and Memmolo^9^. In the first DHM investigations, smears but not live sperm or immobilized sperm cells were observed^8–12^. Additionally, recently, this novel holographic method was used to identify morphometric profiles of live human spermatozoa^7,10,13^ and their motility parameters^14^. Many different variations of phase microscopes have been created^6^. In parallel, inferometric phase microscopy (IPM) with optical imaging based on digital holographic microscopy has been introduced as a mean to investigate human spermatozoa^12,15^. The first attempt to simultaneously assess human spermatozoa using morphology, motility and quantitative stain-free inferometric imaging analysed with a deep-learning algorithm has already been presented^16^.

Another approach used in sperm morphology investigations was to look for negative biomarkers, i.e., proteins and ligands with unique expression on defective spermatozoa. Negative biomarkers can complement the assessment of sperm morphological phenotypes or can be independent factors of their diagnostic evaluation^3^.

Sperm morphology is the most complex and difficult parameter to investigate. The panel of abnormal sperm morphology parameters is broad and can be generally divided into head, midpiece and tail defects. Abnormal sperm morphology – teratozoospermia refers to < 4% normal spermatozoa in the semen sample according to Kruger’s strict criteria^17^. The conventional evaluation of sperm morphology requires the preparation of smears (drying, fixation and staining) and light microscopy. There are many negative aspects of this method: overstaining, artefacts from smearing, staining, and fixation^18^. The development of the computer-assisted sperm analysis of morphology can help operators to assess morphology, but all problems with smear preparation remain the same, and morphological observations are possible only in two dimensions (2D)^19^. This conventional method is still recommended by the World Health Organization (WHO) as a part of routine semen analysis^17^.

The selection of spermatozoa for assisted reproductive therapy/technology (ART) based purely on sperm morphology remains problematic, particularly when the operator must choose only a single spermatozoon for fertilization. By means of MSOME, morphological defects can be identified in vivo, and then single cells can be used immediately for assisted fertilization. This novel method is called intracytoplasmic morphologically selected sperm injection (IMSI). MSOME, however, is still not used as part of routine semen assessment. During the simplest method of ART, intrauterine insemination or conventional IVF (in vitro fertilization), the selection of morphologically normal spermatozoa can be fulfilled by “swim-up” or gradient methods of separation.

The aim of our study was to use the holographic method to investigate the status of sperm morphology and motility in fertile and infertile men using intact live spermatozoa directly after semen liquefaction. Our protocol has many advantages because we can investigate sperm morphology not altered by the process of fixation and staining. We also use DHM to measure all spermatozoa recorded on a given hologram. This means that through the post-factum computer reconstruction of images, we can measure the parameters of sperm, which at the same time change in the sample at different depths. This is not possible by conventional microscopy, with which only the objects in the plane of focus can be properly measured. Moreover, we introduced new morphological parameters of spermatozoa that can be measured in 3D and checked the utility of these parameters for sperm morphology screening.

The technique most frequently explored in andrology laboratories is sperm separation used to obtain the most functional spermatozoa was also assessed by DHM. Some studies have indicated that gradient centrifugation methods should be used, but others have indicated that the “swim-up” technique is the better choice. Both sperm preparation methods allow sperm populations with a low percentage of morphologically abnormal cells to be obtained. Thus the second goal of the study was to assess sperm cell morphology using DHM after “swim-up” and Percoll purifications with the mutual comparison of these two approaches. This is the first study to compare the effects of different sperm separation techniques on sperm morphology using digital holography microscopy in live scenario.

## Results

### Standard semen parameters of fertile volunteer and infertile patients

The parameters measured using conventional light microscopy and evaluated by the conventional method according to the WHO manual are shown in Table 1 and Supplementary Table S1. The specific sperm morphology and calculated TZI (teratozoospermic index) for all participants included in the study are shown in Supplementary Table S2. For the group of healthy volunteers, sperm concentration, the total number of spermatozoa, progressive motility, and the percentage of spermatozoa with normal morphology based on Krűger’s strict criteria were as follows: 82 ± 37 × 10^6^/ml (median ± MAD), total of 260 ± 200 × 10^6^ per sample, 58 ± 17% with progressive motility and 6 ± 5%, with good morphology, respectively. The mean TZI was measured as 1.31 ± 0.17. For the group of infertile males, the mean sperm concentration was 79 ± 36 × 10^6^/ml, the total spermatozoa number in ejaculated sample was 190 ± 130 × 10^6^, progressive motility was measured as 33 ± 18% and normal morphology was measured as 2.5 ± 1.3%. The mean TZI was calculated as 1.45 ± 0.12.

**Table 1:** Descriptive statistics and comparison of standard (WHO) semen parameters in analyzed fertile and infertile men.

	Concentration (× 10^6^/ml)	Number of spermatozoa (× 10^6/^/ejaculate)	Progressive motility (%)	Normal morphology (%)	TZI (teratozoospermia index)
**Fertile (n = 10)**					
Mean ± SD	84 ± 50	330 ± 300	53 ± 20	8.2 ± 6	1.31 ± 0.17
Median	82	260	58*	6**	1.34
MAD	37	200	17	5	0.14
Min–max	21–170	74–840	22–80	3–19	1.09–1.54
25–75%	49–109	152–330	38–64	5–10	1.16–1.46
Skewness	0.52	1.38	− 0.24	1.10	0.04
Kurtosis	− 0.09	0.66	− 1.16	0.35	− 1.64
**Infertile (n = 12)**					
Mean ± SD	78 ± 50	190 ± 130	33 ± 18	2.5 ± 1.3	1.44 ± 0.16
Median	79	150	34 *	3 **	1.45
MAD	36	100	14	1	0.12
Min–max	15–199	52—385	12—72	0—4	1.24–1.79
25–75%	41–94	84–253	18–40	1–3	1.32–1.49
Skewness	1.09	0.08	0.90	− 0.58	0.79
Kurtosis	2.23	− 0.86	0.84	− 0.61	0.97

Statistically significant differences were observed for progressive motility in fertile vs progressive motility in infertile: *P < 0.03; normal forms in fertile vs normal forms in infertile: **P < 0.0008.

Semen parameters of the studied male groups indicate that the sperm count, progressive motility and morphology values were significantly higher in fertile men than in infertile men. Statistically significant differences between the fertile and infertile groups were found for sperm progressive motility (*P* < 0.03) and morphology (*P* < 0.0008).

#### Conventional semen morphological parameters of fertile volunteers and infertile patients

A detailed spermatozoa morphological analysis was performed for all participants (Supplementary Table S2). No statistically significant differences were observed for the TZI index between the fertile and infertile groups.

### Morphological profiles of spermatozoa from fertile and infertile individuals evaluated by DHM

**Figure 1:**
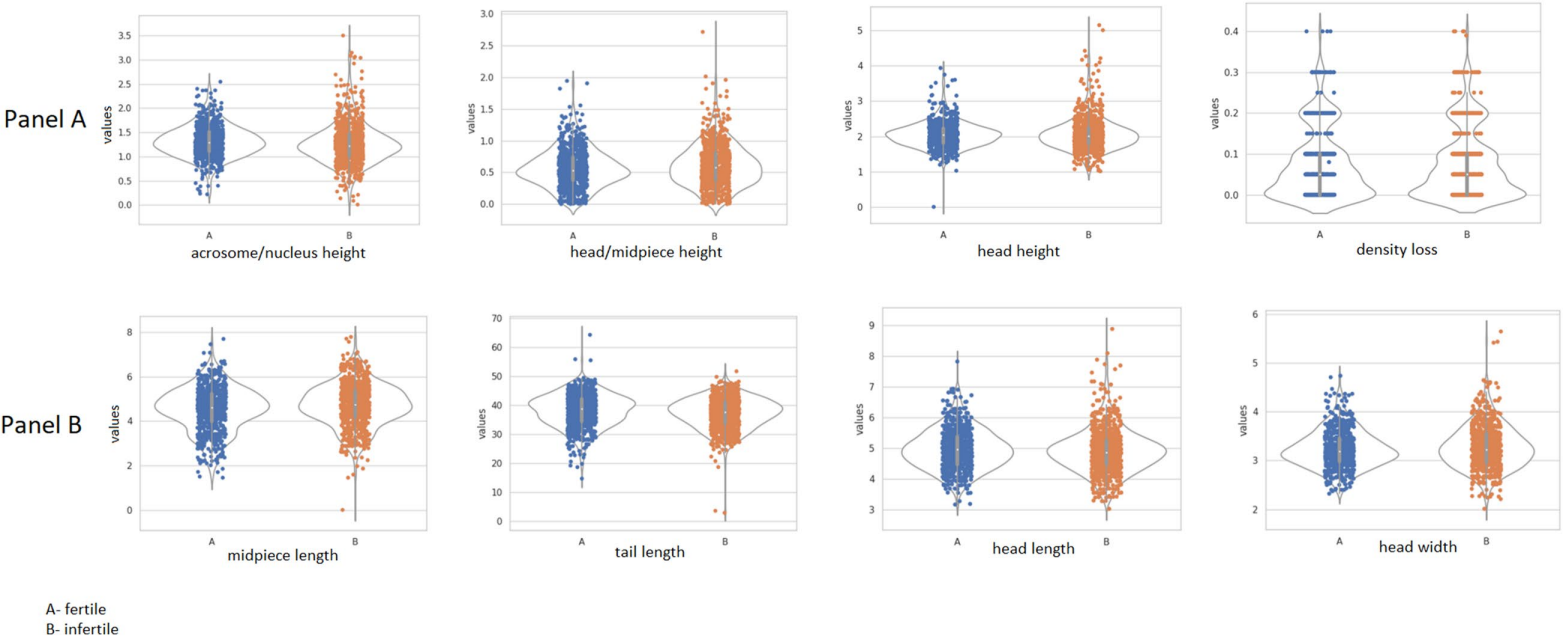
Comparison of spermatozoa 2D and 3D morphological parameters in fertile and infertile men by using DHM (digital holographic microscopy). Panel I (3-D parameters), Panel II (2-D parameters).

Sperm morphological parameters obtained by DHM are presented in Fig. 1 and Supplementary Tables S3, S4, S5, and S6. The median values of sperm morphological parameters obtained from DHM measurements did not differ in a statistically significant fashion for the subgroups investigated (10 fertile vs 12 infertile men). The median midpiece length *(ml)* was slightly lower in the fertile men, and the tail length *(tl)* was slightly lower in the infertile men. However, in the group of infertile men, we observed slightly greater variability in conventional sperm parameter head length *(hl)*, head width *(hw)*, midpiece length *(ml)* and tail length *(tl)* than in the fertile men (see Supplementary Tables S3 and S4 and Supplementary Fig. S1). The results for novel DHM parameters, head height *(hh)*, acrosome/nucleus height *(anh)*, and head midpiece height *(hmh)* are presented in Fig. 1 and Supplementary Tables S5 and S6. The head/midpiece height *(hmh)* was slightly higher for the infertile men, and the acrosome nucleus/height *(anh)* was slightly higher for the fertile men. However, the differences were not statistically significant.

For the parameters measured, we performed the Shapiro–Wilk normal distribution test (*P* > 0.05). Our results indicate that only 25% of values from the infertile group passed the normality test for the *hh* and *anh* parameters, whereas for the fertile group, 45% and 73% of the values passed, respectively. For the parameters obtained by conventional semen analysis, a normal distribution occurred much more frequently in both studied groups, and relative differences in observed percentages between the infertile and fertile groups were smaller.

**Figure 2:**
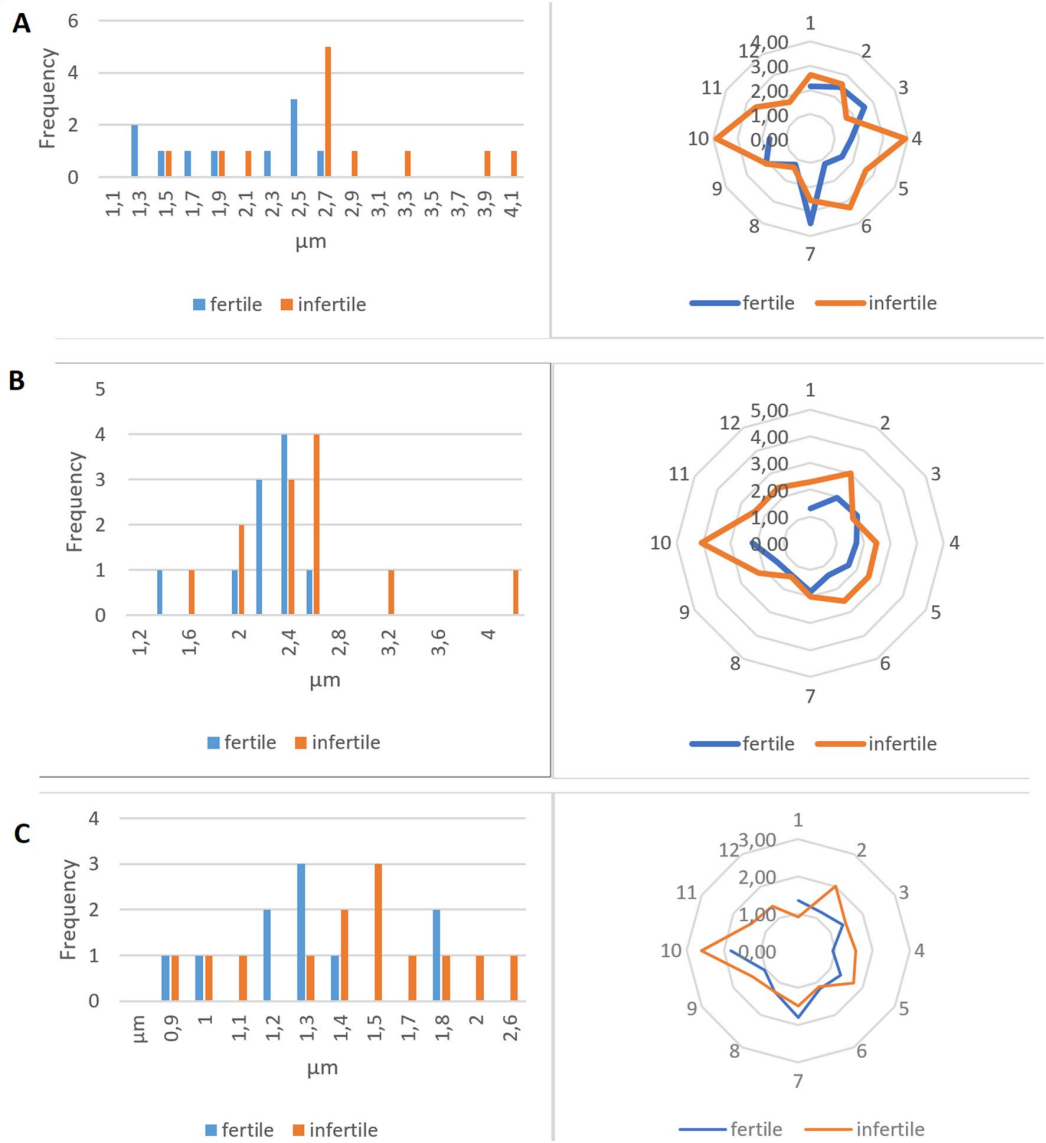
Data range of novel spermatozoa parameters measured by DHM for fertile and infertile men. (**A**) Range of values for *hh* in fertile men (median-1.90 µm) narrowed down in a statistically significant manner compared to the infertile group (median-2.61 µm) (*P* < 0.05). (**B**) Range of values for *amh* (acrosome/nucleus height) narrowed down in a statistically significant manner for the group of fertile men (median- 1.69 µm) vs. infertile individuals (median- 2.34 µm) (*P* < 0.002) (**C**) The median of *hmh* for fertile men was 1.30 µm while that for infertile individuals was – 1.47 µm.

The comparison of the mean value range of the studied sperm parameters for fertile and infertile men shows that for the fertile men, the *hh, anh, hmh* parameters were less variable than those for the infertile men. The results are presented in Table 2. An illustration of these results is presented by the range comparison given in Fig. 2. Narrower ranges of the values from novel DHM parameters were much more frequently observed in the fertile men. We also calculated the mean range for both studied subgroups. The mean range of head height for infertile men was 2.57 µm and that for fertile men was only 2.00 µm, and their percentage ratio was 75.5%. The fertile vs. infertile percentage ratio of the *anh* mean range was 73%, and that for the *hmh* mean range was 77% (see Table 2).

**Table 2:** Mean values of sperm parameters measured by DHM for fertile and infertile men. Significance values are in Bold.

Sperm morphological parameters	WHO (2-D parameters)				DHM (3-D parameters)		
	*hl*-head length (µm)	**hw-head width (µm)**	*ml*-midpiece length (µm)	*tl*-tail length (µm)	*hh*-head high (µm)	*hmh*-head/midpiece high (µm)	*anh*-acrosome/nucleus height (µm)
Fertile individuals	4.96	3.24	4.54	38.22	**2.05**	**0.57**	**1.32**
% of normal distribution in fertile individuals (Shapiro–Wilk)	73%	73%	82%	73%	**45%**	**36%**	**73%**
Infertile individuals	4.91	3.27	4.73	37.26	**2.07**	**0.61**	**1.27**
% of normal distribution in infertile individuals (Shapiro–Wilk)	50%	58%	67%	75%	**25%**	**50%**	**25%**
Mean range ratio fertile vs infertile individuals (%)	85%	79%	87%	91%	**75.5%**	**77%**	**73%**
Fertile vs infertile individuals (Wilcoxon Test) (*P*-value)	0.0999 *P* > 0.05	0.0165 *P* < 0.05	0.3751 *P* > 0.05	0.0883 *P* > 0.05	**0.0226** ***P* < 0.05**	**0.0194** ***P* < 0.05**	**0.0009** ***P* < 0.001**

This finding indicates that the mean values of the *hh*, *anh*, and *hmh* parameters for fertile men are more concentrated than those for infertile men. This tendency was less visible in the case of 2-D morphological parameters, for which such ratios were markedly higher at 79–91% (see Table 2).

### Other DHM sperm traits: motility and density loss, vacuole presence, and head type

DHM enables the morphological assessment of sperm cells in motion. All morphological measurements were performed together with motility type recognition (progressive, nonprogressive, and immotile sperm). The motility profiles of analysed spermatozoa showed good agreement with motility assessed during standard semen analysis.

An optical density loss value > 20% was found for fertile and infertile men with worse morphology when assessed by the conventional Papanicolau method^8^. Optical density loss corresponds to material density loss within the sperm head. Without additional experiments, we cannot determine whether *dl* is caused by less condensed DNA or cytoplasmic/membrane loss. During sperm purification in most cases *(dl),* we observed a statistically significant decrease, especially after the 90% Percoll gradient procedure (Table 3). Our study of the presence of sperm head vacuoles showed a strong correlation between the presence of vacuoles and the type of sperm motility. Few vacuoles were observed in spermatozoa with a high rate of progressive motility.

**Table 3:** Statistical differences of parameters between “native” and separated semen samples by Percoll gradient and “swim-up” technique.

Fertile indivi-duals		Head length-*hl*	Head width-*hw*	Midpiece length-*ml*	Tail length-*tl*	Head height-*hh*	Head/midpiece height- *hmh*	Acrosome/nucleus height-*anh*	Density loss	Progressive motility
No 1	N/S30	0.0001								
	N/S60	0.0000	0.0004		0.0003	0.0001		0.0191	0.0097	0.0000
	N/P47				0.0198			0.0066		0.0000
	N/P90	0.0000			0.0283				0.0097	
No 2	N/S30						0.0002		0.0182	0.0000
	N/S60	0.0067					0.0029			
	N/P47						0.0014			0.0000
	N/P90	0.0005	0.004				0.000		0.0297	
No 3	N/S30	0.0025	0.0010		0,0248					
	N/S60							0.0191		
	N/P47	0.0433	0.0067			0.0001		0.0067		0.0000
	N/P90				0.0012				0.0273	
No 4	N/S30	0.0031								
	N/S60		0.0425							
	N/P47		0.0203				0.0085			
	N/P90		0.0404			0.0000		0.0004		
No 5	N/S30									0.0000
	N/S60			0.0554		0.0241				
	N/P47			0.0208		0.0006	0.0000			0.0000
	N/P90				0.0301				0.0195	
No 6	N/S30									0.0000
	N/S60									0.0000
	N/P47									
	N/P90				0.0020					0.0000
No 7	N/S30									0.0000
	N/S60									
	N/P47									0.0000
	N/P90									0.0248
No 8	N/S30					0.0000		0.0094		0.0070
	N/S60					0.0000		0.0090		0.0070
	N/P90					0.0000		0.0094		0.0275
No 9	N/S30				0.0023					0.0000
	N/S60				0.0000	0.0238				0.0129
	N/P47									
	N/P90						0.0000			

Results are expressed as *P*-value using the Kruscal-Wallis test with Holm adjustment for multiple comparisons.

### Spearman order rank correlations of sperm morphological parameters in the studied subgroups

Table 4 shows the correlations between the values of the DHM parameters studied in the fertile and infertile men. We did not observe any strong correlations (Rs = 0.9–1) but found several moderate and weak ones. Stronger correlations were found for the fertile men. Very weak correlations between the two-dimensional (*hl, hw, ml* and *tt*) parameters were observed. The obtained Rs values indicate rather weak correlations. The novel DHM parameters related to head height did not show strong correlations with the two-dimensional sperm parameters; however, in both tested groups, head height *(hh)* and head length *(hl)* correlated with each other (Rs = -0.389 in the fertile group and Rs = -0.363 in the infertile group). Generally, this correlation was found to be stronger for fertile men than for infertile men. For the group of infertile men, we did not observe any strong correlations.

**Table 4:** Spearman order rank correlation between mean values of morphological sperm parameters obtained by DHM in the studied subgroups.

	R_S_	*P* level
**Variables-fertile group**		
Head height (*hh)* & head length (*hl*)	− 0.389	< 0.001
Head height (*hh)* & acrosome/nucleus height (*anh)*	0.567	< 0.001
Head height (*hh*) & head/midpiece height (*hmh*)	0.333	< 0.001
Head length (*hl*) & volume loss	0.342	< 0.001
Acrosome/nucleus height (*anh*) & head/midpiece height (*hmh*)	0.290	< 0.001
**Variables-infertile group**		
Head height (*hh*) & head length (*hl*)	− 0.363	< 0.001
Head height (*hh*) & acrosome/nucleus height (*anh*)	0.542	< 0.001
Head height (*hh*) & head/midpiece height (*hmh*)	0.381	< 0.001

### Morphological profiles of spermatozoa from fertile volunteers after sperm separation technique application

For fertile volunteers (n = 9), intact sperm and separated spermatozoa were evaluated by the “swim-up” technique and Percoll gradient preparation using a digital holographic microscope. A statistical comparison of sperm morphology parameters showed differences between the samples depending on the method of sperm separation used (30-min swim-up, 60-min swim-up, Percoll 47%, and Percoll 90% sperm fractions). We compared the data of the above mentioned separation methods to the “native” sperm data. The results are presented in Table 3 and are expressed as p values calculated using the Kruskal–Wallis test with Holm adjustment for multiple comparisons. Any single WHO or DHM parameter is enough to distinguish spermatozoa before and after separation. The sperm preparations reveal individual characteristics according to the studied morphological parameters. It may be somewhat useful, but not sufficient, to take into account progressive motility (see the last column of Table 3). The comparison of different methods of sperm selection is presented in Supplementary Table S7, and the results are expressed as p values calculated using the Kruskal–Wallis test with Holm adjustment for multiple comparisons. The comparisons depend on the selection of the semen sample. Individual differences were most often related to 60-min ‘swim-up' and Percoll 90% preparations.

## Discussion

By means of digital holographic microscopy, we investigated 2-D standard sperm parameters as done in conventional 2-D morphometry: head length (*hl*), head width (*hw*), midpiece length (*ml*), and tail length (*tl*). Additionally, we investigated novel morphological parameters characteristic only of the DHM method: head height (*hh*), acrosome/nucleus height (*anh*) and head/midpiece height (*hmh*). Among the studied parameters statistically significant differences were not found. Our groups of fertile and infertile men differed in motility (see Table 1), and the tail and midpiece play an important role in maintaining motility parameters. The median tail length was slightly longer in the fertile men, and the median midpiece length was slightly shorter. The longer tail may correspond with motility^20,21^ but not with the speed of spermatozoa^20^. The length of the midpiece has been shown to correlate with reproductive traits in humans^22^, boars^23^ and other species^21^. On the other hand, a longer midpiece does not denote a well-organized mitochondrial sheath. Short midpieces may contain the highest concentrations of ATP, as proven for birds^24^. The explanation for the observed differences seems to be rather complicated.

The differences in the parameter distributions of fertile and infertile males were found from the Shapiro–Wilk normality test. All novel DHM parameters had a normal distribution in most of the fertile men analysed. The percentage of normal distributions in five of seven morphological parameters was greater in the fertile group. It was especially visible for two new parameters *(hh)* and *(anh),* for which the ratios for fertile vs. infertile were 45%/25% and 73%/25%, respectively. The normality of these parameter distributions could be an additional criterion for distinguishing between fertility and infertility status. The head/midpiece height *(hmh)* did not follow such a rule in most cases. Therefore, the connection between the head and midpiece is more heterogeneous than other DHM parameters.

The nonparametric statistical comparison of three-dimensional parameters *(hmh)* and *(anh)* in fertile and infertile men did not show statistical differences. The head/midpiece height in fertile men was slightly lower, and the acrosome/nucleus height was slightly lower too. The higher *(anh)* is correlated with the larger acrosome area, and this parameter may be important for fertilization. During the “swim-up” procedure, the obtained fraction of selected spermatozoa was enriched with increased acrosome area cells^25,26^.

The most visible difference was found during the analysis of the range of values for clusters of measured parameters. A wider range of values *(hh*, *anh and hmh)* was observed in the group of infertile men. When we compared ranges of the novel DHM parameters connected to the 3-D dimensional analysis *(hh, hmh* and *anh)*, they statistically differed between the fertile and infertile groups. Of the 2-D standard parameters, only the range of head width *(hw*) values differed statistically between the fertile and infertile individuals. Similar volumetric and magnitude observations were made for infertile men with chromosomal changes. The nuclear arrangement of chromosomes can be altered in men with pathological spermiograms or increased DNA fragmentation and in males with chromosomal numerical or structural aberrations. Investigations of infertile men with small supernumerary marker chromosomes (sSMCs) reveal that the chromosome topology in the sperm nuclear territory may lead to infertility^27^. Individual patterns of chromosomal topology may be the result of reciprocal chromosomal translocation^28^. We can only speculate that certain differences in chromosome topology may influence sperm 3-D morphology. On the other hand, vacuoles in spermatozoa can also change chromosomal topology, even when translocation or chromosomal abnormality is not observed^29^. Aziz and colleagues analysed video images of live sperm. The median values of the four morphometric features of the sperm head in semen and sperm preparations were inefficient in discriminating between the two observed groups (fertile and infertile). The authors found a significant difference in sperm head size distribution between these two groups, with fertile males displaying more uniformity in the sperm head area and major axis in both native semen and swim-up preparations than infertile males^30^.

An analysis of Spearman rank order correlations within the morphological parameters obtained by means of DHM in fertile and infertile males was carried out. Generally, more correlations were found in the fertile group than in the infertile group. The morphometric parameters should be closely correlated without marked large differences in measurements of sperm in the fertile male group. On the other hand, the lack of strong correlations can be explained by the fact that even in fertile males, the range of morphologically altered parameters may be high. This is illustrated by the diversity of morphological parameters obtained using the classical Papanicolau method presented in Table 3. Most of the correlations are related to DHM parameters.

We also decided to use the two fractionation methods and check the sperm morphology by DHM. Sperm morphology after separation has never been investigated with the use of live sperm (to our knowledge), except for the use of MSOME for individual spermatozoa during assisted reproduction^4^. For each fertile patient, (n = 9) the morphology of intact sperm and the morphology of separated spermatozoa after “swim-up” and Percoll gradient centrifugation were evaluated. Additionally, we chose two time intervals for the “swim-up” method: 30 and 60 min of incubation. Koyun et al. published a study in which the extension of time for sperm preparation up to 60 min caused a decline in the number of sperm and their progressive and total motility^31^. Sperm separation allows the elimination of leucocytes, immature or damaged spermatozoa, other spermatogenic cells, infectious agents, epithelial cells, etc.^32^. Our study is the first to compare the effects of gradient-density centrifugation and “swim-up” techniques on sperm morphology using DHM for live sperm morphology evaluation. According to the literature, selected spermatozoa should in some way improve their morphology^33–35^. In our study, statistically significant differences were found in the distribution of the morphological parameters of individual spermatozoa. These differences were very subtle, sometimes involving only one parameter. In our experiments, we included ejaculated samples from fertile men with good quality and normal morphological parameters previewed by the conventional Papanicolaou method and with statistically higher TZI values than those of infertile men.

The comparative study of two sperm selection techniques showed that the gradient centrifugation (Percoll 90%) and 60-min “swim-up” methods were the most effective selection methods. More pronounced statistically significant differences were found when using Percoll gradient centrifugation. Prakash et al.^35^ presented similar results obtained by conventional sperm morphology assessment (light microscopy and sperm strict criteria). The authors found gradient centrifugation and “swim-up” methods to be comparable in their effects on specific sperm elimination abnormalities and that after Percoll, a significantly larger number of samples with good sperm morphology can be obtained^35^. The main principle of both selection methods is based on the fact that immotile spermatozoa with poor morphology are selectively removed, which increases the percentage of motile spermatozoa with good morphology. Digital holographic microscopy might be helpful in selecting the most effective semen separation method for individual patients.

Swim-up selection after 60 min of incubation was more effective than after 30 min. The time of incubation may be crucial for spermatozoa, since male germ cells are temperature sensitive. The enrichment of high-quality sperm samples over the shortest time period possible is the goal of all andrological laboratories. A shorter incubation time (30 min) would have been more feasible but was ineffective for the morphological selection of spermatozoa we investigated. “Swim-up” and Percoll gradient centrifugation have been regularly used and seem to be the most popular techniques for sperm selection. Both procedures are based on the motility of sperm, which does not mean that all selected sperm are of the highest quality^36^. These selection techniques are probably not efficient in selecting spermatozoa with respect to apoptosis, DNA integrity, cell membrane maturation and sperm ultrastructure^37^. The results obtained from our experiments by the DHM technique clearly show that the morphology of spermatozoa obtained after adopting selection methods is not “perfect” in many cases.

Very recently, Ben-Yehuda et al.^16^ presented a new technique for the live sperm analysis of individual unstained live cells. The authors measured DNA fragmentation, morphology with virtual staining and motility. In our study, we analysed sperm morphology and motility in individual unstained live cells without DNA fragmentation and virtual staining. Ben-Yehuda et al.’s method is based on the use of quantitative stain-free interferometric phase imaging and deep-learning frameworks, with which the authors were able to perform the morphological evaluation, motility and DNA fragmentation of the same cell^16^. The authors used 8 randomly selected donors, but we have distinguished two groups of males: 10 fertile and 12 infertile. Our groups of men were also selected randomly, and we were surprised that our small groups of investigated men differed statistically in classical morphology evaluation and motility (Table 1). Such results indicate that morphology and motility are two of the most powerful fertility evaluation parameters, and classical fertility evaluations should focus on them. Our work differs in the study of morphology. Ben-Yehuda et al.^16^ used virtual staining and investigated parameters such as nucleus area, acrosome area, total head area, mean posterior-anterior difference, and dry mass. In our work, we distinguished 2-D parameters, including head length, head weight, and midpiece and tail length, and 3-D parameters, including head height, acrosome/nucleus height and head/midpiece height. In our work, we measured the morphology of intact spermatozoa in seminal plasma droplets and then separated them in two distinct ways (swim-up and Percoll gradient separation). Ben-Yehuda et al. used spermatozoa after gradient separation only. Both studies could make an important contribution to research on the sperm morphology on living cells.

The results obtained by DHM may be important for patients undergoing different assisted reproduction protocols and may be a validating procedure to indicate the efficacy of different methods of sperm selection. Spermatozoa are highly heterogeneous cell populations even in fertile ejaculated samples, and methods for their optimization may be highly valued in ART clinics. The cost of DHM equipment is similar to that of high-quality classical microscopes and will be no obstacle to specialty centres for reproductive investigation. Despite the cost of using a microscope with professional software, the preparation of samples generates no additional costs. This technology is ready to be fully utilized in clinical laboratories.

In summary, the classical Papanicolau morphology examination showed a statistically significant difference between our fertile and infertile groups, while the measurements with DHM did not. Interestingly in the classical method there was no difference in TZI. In the context of a reduced WHO standard to 4% of good morphology the huge heterogeneity of remained sperm may account for the lack of variation except for such characteristic defects as e.g. globozoospermia. On the other hand more accurate morphology studies by DHM allow more defects to be examined and the difference between normal and abnormal ejaculate would become blurred. This in our view, does not compromise a search for optimal live sperm morphology evaluation which still awaits to be discovered.

## Material and methods

### Cohort recruitment

Infertile patients and fertile volunteers were invited through local advertisements to take part in the study. Volunteers were fertile men whose female partners had conceived within 12 months before the donation of semen samples to this study. Semen samples from 10 fertile and 12 infertile men with an average age of 33 ± 6 years were investigated. Semen samples (n = 22) were obtained by masturbation after 3–4 days of sexual abstinence. One sample per each patient/volunteer was used for the study.

### Semen analysis

All semen samples underwent liquefaction at room temperature and were evaluated within one hour of collection via standard light microscopy according to the guidelines of the World Health Organization^17^. Conventional semen parameters such as sperm count, motility, viability and morphology were assessed.

### Papanicolau staining

After preliminary semen evaluation, two slides of semen smears were prepared from each semen sample. Sperm samples were centrifuged (600 g, 7 min), and sperm pellets were rinsed once in prewarmed PBS to 37 °C (600 g; 2.5 min). Cell pellets were resuspended in warm PBS (37 °C), and 10 µl of sperm suspension was spread onto a slide. The appropriate sperm density was visually inspected and controlled by light microscopy. The air-dried smears at room temperature were fixed in a solution of ethanol and diethyl ether (1:1 ratio, v/v) for 10 min and then air-dried again. Then, slides were stained by the Papanicolau staining procedure and mounted in DPX Mountant (Sigma Aldrich, Saint Louis*,* MO, USA). A total of 200 spermatozoa were observed under × 1000 magnification and oil immersion. The sperm sample was assumed to be normal when 4% of spermatozoa with good morphology according to Kruger’s strict criteria were present. All microscopic slides were prepared by the same person using the same technique. Papanicolau staining is one of the three main methods recommended by the WHO^17^.

### Sperm selection methods

Semen samples from healthy, fertile volunteers were divided into three parts, and three different fractionation methods were used: 30 min of “swim-up” incubation, 60 min of “swim-up” separation and Percoll gradient centrifugation.

### “Swim-up” method

The investigation was carried out on spermatozoa prepared by the ‘swim-up’ technique for the selection of the most motile, viable and morphologically normal fraction of spermatozoa. The sperm sample was rinsed with a medium consisting of Ham’s F-10 supplemented with 1% BSA (bovine serum albumin) by centrifugation. After the final rinse, 2 ml of medium was gently overlayered over the sperm pellets. For every semen sample, two tubes were prepared. One was incubated for 30 min, and the second was incubated for 60 min at 37 °C in 5% CO2 humidified air. The isolated spermatozoa were collected, washed in F-10 medium and resuspended to a concentration of 40 × 10^6^/ml. The percentage of sperm with good morphology and abnormal forms was then compared during pre- and postswim-up sampling by DHM.

### Percoll density gradient centrifugation

Spermatozoa were isolated via the density gradient centrifugation of Percoll at 90% and 47%. For the lower layer, 2 ml of 90% Percoll was transferred to a conical centrifuge tube. For the upper layer, 2 ml of Percoll 47% was transferred onto the top of gradient. After liquefaction, the third part of the semen sample (0.6–2 ml) was dispensed onto the top. The tube was centrifuged for 30 min at 430 g^17^. The concentration and motility were recorded, and fractionated spermatozoa were resuspended to a concentration of 40X10^6^/ml.

### DHM procedure

We used a DHM T1000 digital holographic transmission microscope in an off-taxis Mach–Zehnder configuration^38^. A 666 nm laser diode with optical fibre coupling for enhanced stability, illuminating the sample with very little power (down to 100 µW/cm^3^), was used. The sperm samples were observed with a magnification of 50 × using a dry objective/condenser assembly (NA = 0.75, FOV-104 µm). The hologram was registered with a CCD camera with 1024 × 1024 pixels at 15 fps. From a single recorded hologram, the image of the sample was reconstructed numerically^39^. The Lyncée Tec DHM was delivered through Koala software, which allowed for the measurement and interpretation of the data obtained^38^.

To assess the morphometric characteristics of spermatozoa, a 10 µl of fresh semen sample or spermatozoa after separation (swim-up or Percoll) was loaded onto a clear glass slide, covered with a 22 × 22 mm^2^ cover glass, and observed under a holographic microscope at a magnification of 50x. The final sperm concentration needed to be no more than 40 × 10^6^/ml. If the sperm concentration was higher, we diluted the semen samples by seminal plasma. Seminal plasma was used to maintain motility and to not disturb the native sperm environment.

The morphology of spermatozoa from fertile and infertile men by the DHM technique was evaluated directly after semen liquefaction. Spermatozoa from fertile volunteers were investigated following a “swim-up” period of 30 min (S30), “swim-up” of 60 min (S60), Percoll 90% (P90), and Percoll 47% (P47) gradient preparation.

Sixty spermatozoa from each sperm sample were examined for the morphological status of critical parameters: the head length *(hl)*, midpiece length *(ml)*, tail length *(tl)*, head width *(hw)*, head height *(hh)*, acrosome/nucleus height *(anh)*, and head/midpiece height *(hmh)*.

#### Holographic imaging of spermatozoa; 3D parameters

Using DHM T1000 and Koala software, we were able to measure new 3D parameters:

*head height (hh)* – the maximum height of spermatozoon.

*acrosome/nucleus height (anh)* – the height of spermatozoon where the acrosome becomes visible.

*head/midpiece height (hmh)* – the height of spermatozoon at the point where the midpiece is connected and *density loss (dl) or volume loss (vl)* (a loss of sperm optical density visible as a loss of shape on the cross section) and defined in %.

We also investigated the *head length (hl)* (the length of the head on the longest axis in µm); *head width (hw)* (the width of the head at the widest point in µm), *midpiece length (ml)* (the length of the midpiece on the longest axis in µm); *tail length (tl)* in µm; *head type* (the pattern of the shape of the sperm head after the passage of the laser beam); and *presence of vacuoles*. At the same time, we registered a motility to recognize frame by frame progressive motility, nonprogressive motility and a lack of motility.

**Figure 3:**
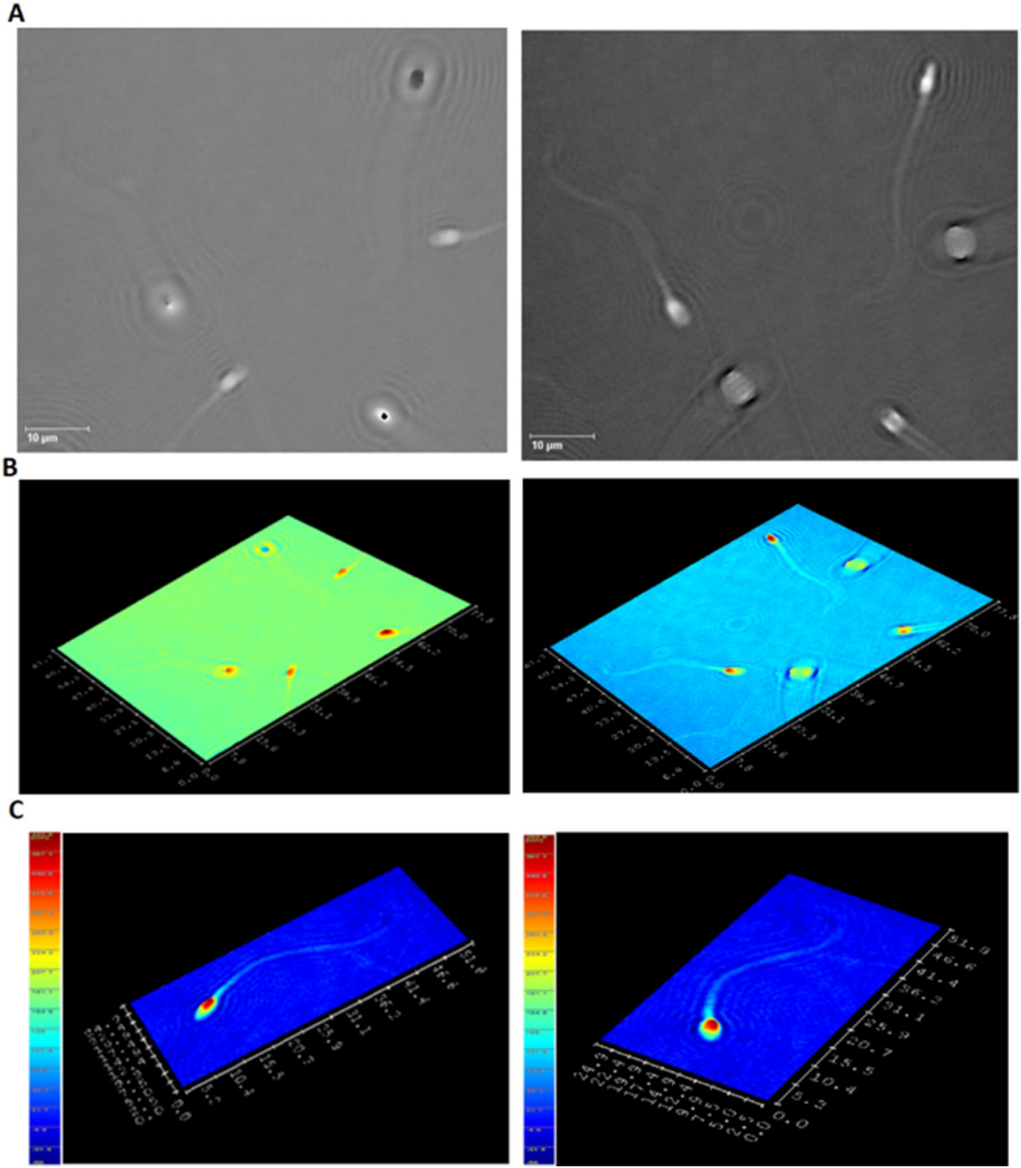
Holographic imaging of spermatozoa obtained via DHM. (**A**) Two phase images based on one hologram with sharpness at different sample depths. (**B**) Two computerized 3D reconstructions based on one hologram with sharpness at different sample depths. (**C**) 3D reconstructions of a single spermatozoon.

Figure 3 shows examples of phase images and 3D reconstructions.

Supplementary Figures S2–S8 present sample measurements of the morphological parameters.

### DHM quality control

Personal intervariability was not assessed because the entire study was performed by one experienced observer (MK). Additional tests of the DHM T1000 phase images were performed using sperm samples collected from the same individual. We compared the images obtained for the sperm cells in the semen smear or stained with Papanicolau method as well as for the live cells in liquid PBS or seminal plasma. There were no diffraction aberrations with the phase images for the fixed cells (see Fig. S9). The results differed, however, depending on sample preparation (see. Fig. S9 (a) and (b)). For the sperm cells in liquid we see diffraction patterns (see e.g. Fig. S2–S8). Probably smaller differences in the refraction index (between the sperm and liquid medium) could make the numerical reconstruction more difficult. It may affect the measurement uncertainty of the 2D-parameters but not more than for a few percent (see Fig. S10). Additionally, we used the capabilities of the DHM T1000 digital microscope for the simultaneous registration of intensity and phage images. It makes easier to focus the sample or optionally to measure classical morphological parameters using two types of images. The results of the sperm head width measurements from both images are shown in Fig. S9 (fixed cells) and in Fig. S10 (live cells). The results obtained from the intensity and phase profiles did not differ significantly.

Greater measurement uncertainty may be caused by the operator selecting the profile line position or the cursor positions. Digital adjustments of the focus distance in the range considered by the operator in order to give a sharp image cause changes in the width measurement may provide no more than a few percent difference (see Fig. S11a–b). Only the measurements out of focus may differ significantly (see Fig. S11c). A way to handle these focusing problems is described by Rinehart et al.^40^. The effect of the setting up the digital adjustments of the focus distance (f in cm) for the phase image during computer reconstruction was also checked. The change of the setting can affect the values of the new 3D-parameters the most. The optimal focus distance is zero^41^. We found that the change in the distance by ± 0.2 cm (in steps of 0.1 cm, the smallest possible step with the Koala software) does not influence the height measurements much. In this case the operator does not see a significant change in the sharpness of the measured object. A greater range of the f-changes causes differences in the measured values of up to 10 percent (see Fig. S12), however in such case the operator no longer considers the image of the object to be sharp (case (c) in Fig. S12). We concluded, that although it is more difficult to measure sperm morphological parameters of the live sperm than the fixed samples, the measurement uncertainties are small enough in order not to affect the final results of the study.

### Statistical evaluation

A statistical analysis of the data was performed using the STATISTICA software package, versions 10.0 and 13.0 (StatSoft). The Shapiro–Wilk test was used to check the normal distribution of the evaluated parameters. The correlations were calculated using the Spearman rank test. The variation in parameters evaluated before and after density gradient centrifugation was tested by the Wilcoxon signed ranks test. For all statistical tests, differences with a *P* value of < 0.05 were considered significant. The data were also analysed using the Python 3 with pandas (http://pandas.pydata.org), Matplotlib (https://matplotlib.org/), SciPy (https://www.scipy.org/) and scikit-posthoc (https://pypi.org/project/scikit-posthocs/) libraries. Statistical differences among the studied subgroups were determined by the nonparametric Kruskal–Wallis test. For the post hoc analysis, the Dunn test with Holm’s correction was applied.

### Statement of ethical approval

Ethical committee approval (the Local Bioethical Committee of the Poznan University of Medical Sciences, approval no. 771/15) was received for the study. All patients and volunteers gave their written informed consent to participate. All experiments were performed in accordance with relevant guidelines and regulations.

## Conclusion

The DHM method enables more accurate, quantitative and objective morphological measurements of live, unlabelled sperm cells with the simultaneous assessment of the motility of the tested spermatozoa. The main advantage of DHM is the possibility of investigating the entire population of sperm cells in a certain volume of a sample, and not only sperm cells that have been registered in a microscope’s plane of focus. Moreover, 3D parameters provide new information on live sperm morphology that may be helpful in assessing fertility and that might differentiate their fertilization ability with respect to external factors applied.

We can precisely monitor the quality of sperm cells after ‘swim-up’ or Percoll gradient selection. Thus, holography could be used in combination with ART techniques. In assisted reproductive therapy (ART), especially intracytoplasmic sperm injection (ICSI), the operator should select the best motile spermatozoon. The ability to identify potentially fertile spermatozoa in a microscope without destruction is highly desired by embryologists.

## Supplementary Information

Supplementary Information.
