# Supplementary material for: Human live spermatozoa morphology assessment using digital holographic microscopy
Source: Sci Rep. 2022 Mar 22;12:4846. doi: 10.1038/s41598-022-08798-6 (PMC8940907; doi:10.1038/s41598-022-08798-6)

**Supplementary Table S1.** Standard (WHO) semen parameters of analyzed fertile and infertile men.

|  | **Concentration**  **(10^6^/ml)** | **Number**  **of spermatozoa**  **(mln)** | **Progressive motility (%)** | **Normal forms (%)** | **TZI**  **(teratozoo-spermia**  **index)** |
| --- | --- | --- | --- | --- | --- |
| Fertile 1 | 120 | 840 | 36 | 5 | 1.36 |
| Fertile 2 | 105 | 262.5 | 61 | 15 | 1.49 |
| Fertile 3 | 170 | 816 | 76 | 9 | 1.15 |
| Fertile 4 | 47 | 141 | 43 | 19 | 1.13 |
| Fertile 5 | 110 | 264 | 65 | 7 | 1.36 |
| Fertile 6 | 65 | 292.5 | 62 | 3 | 1.49 |
| Fertile 7 | 44 | 110 | 80 | 11 | 1.18 |
| Fertile 8 | 98 | 186.2 | 54 | 5 | 1.09 |
| Fertile 9 | 21 | 73.5 | 22 | 5 | 1.31 |
| Fertile 10 | 56.5 | 339 | 28 | 3 | 1.54 |
| Infertile 1 | 14,8 | 51.8 | 18 | 3 | 1.24 |
| Infertile 2 | 17.0 | 68.0 | 17 | 2 | 1.52 |
| Infertile 3 | 90.0 | 225 | 45 | 1 | 1.48 |
| Infertile 4 | 199.3 | 338.9 | 22 | 3 | 1.43 |
| Infertile 5 | 74.0 | 148,0 | 35 | 2 | 1.48 |
| Infertile 6 | 67.0 | 134.0 | 50 | 0 | 1.60 |
| Infertile 7 | 90.0 | 180.0 | 38 | 4 | 1.26 |
| Infertile 8 | 110.0 | 385.0 | 72 | 4 | 1.28 |
| Infertile 9 | 36.2 | 79.6 | 38 | 4 | 1.33 |
| Infertile 10 | 108.0 | 151.2 | 12 | 3 | 1.79 |
| Infertile 11 | 43 | 86 | 33 | 3 | 1.47 |
| Infertile 12 | 84.3 | 379.5 | 17 | 1 | 1.41 |

**Supplementary Table S2.** Detailed sperm morphological parameters in fertile and infertile men obtain by classical Kruger’s assessment.

|  | | | **Head defects (%)** | | | | | | | | **Midpiece defects (%)** | | | | **Tail defects (%)** | | | |  |
| --- | --- | --- | --- | --- | --- | --- | --- | --- | --- | --- | --- | --- | --- | --- | --- | --- | --- | --- | --- |
| **Patient number** | **M** | **TZI** | **H1** | **H2** | **H3** | **H4** | **H5** | **H6** | **H7** | **H8** | **Mp1** | **Mp2** | **Mp3** | **Mp4** | **T1** | **T2** | **T3** | **T4** | **C** |
| Fertile 1 | **5** | 1.36 | 19 | 5 |  | 5 | 63 | 37 | 5 |  |  |  | 1 |  |  |  | 2 |  | 1 |
| Fertile 2 | **15** | 1.49 | 5 | 7 |  | 13 | 66 | 48 | 6 |  | 1 |  | 1 |  |  |  | 1 |  |  |
| Fertile 3 | **9** | 1.15 |  | 16 |  | 23 | 30 | 36 | 5 |  | 1 |  |  |  |  |  | 2 |  | 3 |
| Fertile 4 | **19** | 1.13 | 2 | 27 |  | 22 | 44 | 20 | 3 | 1 | 2 |  | 5 |  |  |  | 7 |  | 2 |
| Fertile 5 | **7** | 1.36 | 1 | 12 | 1 | 18 | 41 | 32 | 14 | 2 | 1 |  | 3 | 1 |  |  | 9 |  | 5 |
| Fertile 6 | 3 | 1.49 | 21 | 25 | 1 | 20 | 52 | 28 | 3 |  |  |  |  |  |  |  | 2 |  |  |
| Fertile 7 | 1 | 1.18 | 7 | 33 |  | 25 | 31 | 15 | 7 |  | 1 | 1 |  |  |  |  | 2 |  |  |
| Fertile 8 | **5** | 1.09 |  | 1 |  | 6 | 35 | 61 | 2 | 2 |  |  |  |  |  |  | 5 |  |  |
| Fertile 9 | **5** | 1.31 |  | 7 |  | 25 | 31 | 51 | 5 | 2 | 1 |  | 1 |  |  |  | 6 |  | 4 |
| Fertile 10 | 3 | **1.54** | 9 | 12 | 2 | 24 | 37 | 35 | 1 | 1 | 2 |  | 7 |  | 1 |  | 19 |  | 9 |
| Infertile 1 | 3 | 1.24 | 4 | 13 | 1 | 9 | 23 | 54 | 3 |  |  |  | 5 |  |  |  | 6 |  | 9 |
| Infertile 2 | 2 | **1.52** | 1 | 15 | 1 | 18 | 57 | 24 | 8 |  | 4 |  | 2 |  |  |  | 11 |  | 12 |
| Infertile 3 | 1 | 1.48 | 6 | 27 |  | 26 | 49 | 22 | 4 | 2 | 1 |  | 1 |  |  |  | 10 |  | 3 |
| Infertile 4 | 3 | 1.43 | 6 | 32 |  | 35 | 23 | 11 | 4 | 4 | 2 |  |  |  |  |  | 28 |  | 1 |
| Infertile 5 | 2 | 1.48 | 6 | 1 | 1 | 38 | 35 | 26 | 5 | 1 | 4 |  | 2 | 1 | 1 | 2 | 15 |  | 5 |
| Infertile 6 | 0 | **1.60** | 18 | 6 | 2 | 61 | 22 | 14 | 1 | 1 | 6 |  | 2 | 1 |  | 2 | 22 |  | 6 |
| Infertile 7 | **4** | 1.26 | 3 | 18 | 2 | 28 | 29 | 18 | 21 | 1 | 1 | 1 |  |  | 2 | 1 | 1 |  |  |
| Infertile 8 | **4** | 1.28 |  | 20 |  | 10 | 58 | 37 | 2 |  | 1 |  |  |  |  |  | 1 |  | 1 |
| Infertile 9 | **4** | 1.33 |  | 28 |  | 11 | 38 | 47 | 2 |  | 2 |  | 2 |  |  |  | 1 |  | 4 |
| Infertile 10 | 3 | **1.79** | 8 | 15 |  | 27 | 32 | 28 | 8 |  | 10 | 1 | 5 |  |  |  | 18 |  | 10 |
| Infertile 11 | 3 | 1.47 | 6 | 10 | 5 | 31 | 45 | 26 | 7 |  | 3 |  |  |  |  |  | 11 |  | 2 |
| Infertile 12 | 1 | 1.41 | 7 | 22 |  | 25 | 24 | 33 | 2 | 4 | 3 |  | 4 |  | 1 |  | 8 |  | 8 |

M-% normal morphology, TZI- teratozoospermia index, H – head defects, Mp -midpiece defects, T-tail defects, C- residual cytoplasm head defects: H1- tapered head, H2- pyriform head, H3- small head, H4- amorphous head, H5- vacuolated head, H6- small acrosome, H7- big acrosome, H8-no acrosome

**Supplementary Table S3**. Spermatozoa two-dimensional parameters obtained using DHM in live sperm.

|  | | Fertile men | | | | | | | | | | Infertile men | | | | | | | | | | | |
| --- | --- | --- | --- | --- | --- | --- | --- | --- | --- | --- | --- | --- | --- | --- | --- | --- | --- | --- | --- | --- | --- | --- | --- |
|  |  | F1 | F2 | F3 | F4 | F5 | F6 | F7 | F8 | F9 | F10 | IF1 | IF2 | IF3 | IF4 | IF5 | IF6 | IF7 | IF8 | IF9 | IF10 | IF11 | IF12 |
| Head length (µm)  *(hl)* | Mean  SD  Median  Min  Max  25%  75%  MAD | 5.00  0.7  4.89  3.27  6.93  4.6  5.27  0.5 | 4.48  0.5  4.47  3.55  5.4  4.1  4.84  0.4 | 4.80  0.6  4.74  3.16  6.15  4.45  5.14  0.5 | 4.89  0.5  4.85  3.66  6.66  4.59  5.14  0.4 | 4.65  0.5  4.62  3.79  6.81  4.33  4.87  0.4 | 5.40  0.6  5.49  3.54  6.81  5.06  5.79  0.5 | 5.41  0.7  5.49  3.69  7.82  4.96  5.75  0.5 | 4.44  0.5  4.33  3.53  5.88  4.17  4.76  0.4 | 4.91  0.5  4.85  3.18  6.27  4.57  5.23  0.4 | 5.17  0.6  5.13  3.93  6.90  4.73  5.50  0.4 | 4.71  0.6  4.72  3.54  6.27  4.36  5.08  0.5 | 4.75  0.7  4.65  3.39  7.07  4.29  5.10  0.5 | 5.07  0.7  5.10  3.40  7.46  4.60  5.37  0.5 | 5.18  0.9  5.03  3.55  7.88  4.66  5.64  0.7 | 5.30  0.7  5.30  3.52  7.69  4.96  5.66  0.5 | 5.13  0.8  4.98  3.80  8.88  4.75  5.38  0.5 | 4.87  0.5  4.85  3.28  7.07  4.58  5.15  0.4 | 4.57  0.6  4.57  3.39  6.64  4.20  4.97  0.4 | 4.53  0.5  4.47  3.41  6.07  4.17  4.87  0.4 | 5.24  0.8  5.05  3.52  7.17  4.72  5.82  0.6 | 4.86  0.7  4.86  3.27  6.28  4.47  5.11  0.5 | 4,86  0.9  4.84  3.02  7.57  4.19  5.41  0..7 |
| Head width (µm)  *(hw)* | Mean  SD  Median  Min  Max  25%  75%  MAD | 3.10  0.3  3.1  2.32  4.0  2.94  3.29  0.2 | 3.20  0.3  3.12  2.68  4.01  3.05  3.340.2 | 3.28  0.3  3.32  2.55  3.87  3.08  3.87  0.2 | 3.29  0.3  3.27  2.79  3.99  3.09  3.45  0.2 | 3.16  0.4  3.12  2.52  4.37  2.93  3.37  0.3 | 3.26  0.3  3.24  2.55  4.34  3.07  3.44  0.3 | 3.57  0.5  3.50  2.63  4.74  3.19  3.95  0.4 | 3.31  0.4  3.31  2.53  4.10  3.05  3.580.3 | 3.42  0.4  3.39  2.75  4.38  3.16  3.69  0.3 | 3.11  0.4  3.05  2.37  4.71  2.89  3.30  0.3 | 3.20  0.4  3.19  2.39  4.09  2.99  3.43  0.3 | 3.44  0.5  3.43  2.01  4.61  3.07  4.61  0.4 | 3.35  0.4  3.37  2.26  4.12  3.05  3.69  0.4 | 3.29  0.6  3.24  2.24  5.44  2.95  3.50  0.4 | 3.53  0.4  3.51  2.67  4.65  3.30  3.83  0.3 | 3.13  0.5  3.02  2.55  5.65  2.80  3.23  0.3 | 3.39  0.4  3.34  2.56  4.62  3.13  3.62  0.3 | 3.24  0.4  3.22  2.53  4.19  2.94  3.46  0.3 | 3.02  0.3  2.97  2.27  3.63  2.80  3.25  0.3 | 3.13  0.3  3.18  2.26  4.02  2.95  3.33  0.2 | 3.19  0.4  3.06  2.55  4.57  2.92  3.34  0.3 | 3.13  0.4  3.12  2.21  4.23  2.80  3.43  0.4 |
| Midpiece length (µm)  *(ml)* | Mean  SD  Median  Min  Max  25%  75%  MAD | 5.0  0.8  4.99  2.72  7.07  4.57  5.39  0.6 | 4.82  0.9  4.82  2.28  6.55  4.21  5.50  0.8 | 5.17  0.7  5.29  3.12  6.60  4.80  5.67  0.5 | 4.54  1.0  4.61  2.25  7.70  4.17  5.50  0.7 | 4.98  0.7  4.89  2.75  7.08  4.61  5.37  0.5 | 5,38  0.6  5.35  4.07  6.42  4.93  5.90  0.5 | 4.55  0.8  4.58  2.63  6.27  4.07  4.98  0.6 | 4.40  0.6  4.45  3.14  5.62  4.08  4.83  0.4 | 4.35  0.9  4.31  2.24  6.26  3.79  4.98  0.7 | 3.81  1.0  3.91  1.45  6.70  3.14  4.59  0.8 | 4.89  1.0  4.86  2.61  7.71  4.19  5.52  0.7 | 4.78  1.0  4.66  2.87  7.57  4.18  5.49  0.8 | 4.35  0.8  4.32  2.61  5.99  3.84  4.88  0.6 | 5.07  1.1  5.23  2.64  6.79  4.33  5.72  0.9 | 4.94  0.7  4.99  3.13  6.38  4.61  5.39  0.5 | 4.80  1.0  5.13  2.63  6.77  4.31  5.54  0.8 | 4.73  0.9  4.70  1.86  7.10  4.21  5.34  0.7 | 4.38  0.8  4.33  2.35  6.00  4.02  4.85  0.6 | 4.81  1.0  4.91  2.24  7.79  4.22  5.49  0.8 | 4.79  1.3  4.73  0.0  6.68  4.23  5.7  0.9 | 4.50  0.9  4.59  1.59  7.04  4.05  5.12  0.7 | 4.64  0.9  4.72  2.61  6.44  4.19  5.28  0.7 |
| Tail length (µm)  *(tl)* | Mean  SD  Median  Min  Max  25%  75%  MAD | 37.9  5.1  36.6  26.6  48.9  34.4  41.9  4.3 | 37.3  4.8  37.3  28.1  48.9  34.4  40.8  3.9 | 39.9  4.7  40.1  28.0  55.5  36.9  43.0  3.7 | 37.0  4.4  37,0  26.5  47.7  33.7  40.2  3.6 | 41.5  5.6  43.0  18.7  48.9  37.8  45.4  4.2 | 41.0  3.9  40.8  33.3  49.5  38.0  43.7  3.1 | 38.4  7.3  39.8  14.7  55.9  34.4  42.9  5.6 | 37.0  4.8  37.4  23.046.0  32.9  41.1  4.0 | 37.6  5.1  37.4  22.8  46.6  35.0  40.9  4.0 | 37.1  6.5  38.5  19.2  64.3  32.7  41.9  5.3 | 37.2  4.5  38.5  25.7  46.5  33.7  40.0  3.6 | 35.7  5.7  36.8  25.9  48.2  31.1  39.7  4.9 | 34.8  4.6  34.4  25.7  44.6  31.4  38.0  3.8 | 37.4  4.8  37.5  26.8  45.2  34.4  40.8  4.0 | 36.2  5.03  36.4  26.1  48.3  32.5  38.9  4.0 | 36.9  5.7  37.6  18.7  46.4  33.9  41.6  4.5 | 38.4  4.9  39.1  26.8  47.9  35.1  42.2  4.1 | 38.5  5.2  38.4  27.1  51.7  34.8  41.6  4.2 | 37.6  4.3  37.4  24.7  45.6  35.3  45.6  3.4 | 38.2  5.2  40.0  23.7  47.2  35.6  41.6  4.1 | 36.2  7.7  38.4  2.89  45.3  33.6  40.8  5.2 | 37.1  5.2  37.3  20.8  48.2  34.9  39.5  3.7 |

**Supplementary Table S4.** Descriptive statistics and comparison of spermatozoa two-dimensional parameters obtained in fertile and infertile men by using DHM measurements of live sperm.

|  | Head length (µm)  *(hl)* | Head width (µm)  *(hw)* | Midpiece length (µm)  *(ml)* | Tail length (µm)  *(tl)* |
| --- | --- | --- | --- | --- |
| Fertile (n=10) | | | | |
| Mean ± standard deviation | 4.96 ± 0.7 | 3.24 ± 0.4 | 4.5 ± 1 | 38.2 ± 6 |
| Median | 4.9 | 3.19 | 4.6 | 38.8 |
| MAD | 0.5 | 0.3 | 0.8 | 5.0 |
| Min - max | 3.16 – 7.82 | 2.32 – 4.74 | 1.4-7.7 | 14.7 – 64.3 |
| 25% - 75% | 4.48 - 5.37 | 2.98 – 3.46 | 5.0 – 5.2 | 34.5 – 42.2 |
| skewness | 0.37 | 0.58 | -0.33 | -0.34 |
| kurtosis | 0.47 | 0.65 | 0.17 | 0.91 |
| Infertile (n=12) | | | | |
| Mean ± standard deviation | 4.91 ± 0.7 | 3.27 ± 0.5 | 4.73 ± 1 | 37.26 ± 6 |
| Median | 4.86 | 3.22 | 4.73 | 37.71 |
| MAD | 0.5 | 0.3 | 0.8 | 4.0 |
| Min - max | 3.02 – 8.88 | 2.01 – 5.65 | 0 – 7.79 | 2.89 – 51.73 |
| 25% - 75% | 4.46 – 5.25 | 2.96 - 3.55 | 4.19 – 5.37 | 33.80– 41.0 |
| skewness | 0.83 | 0.73 | -0.26 | -0.84 |
| kurtosis | 2.55 | 1.99 | 0.76 | 3.16 |

**Supplementary Table S5**. Spermatozoa three-dimensional parameters and volume loss obtained using DHM measurements in live sperm.

|  | | Fertile men | | | | | | | | | | Infertile men | | | | | | | | | | | |
| --- | --- | --- | --- | --- | --- | --- | --- | --- | --- | --- | --- | --- | --- | --- | --- | --- | --- | --- | --- | --- | --- | --- | --- |
|  |  | F1 | F2 | F3 | F4 | F5 | F6 | F7 | F8 | F9 | F10 | IF1 | IF2 | IF3 | IF4 | IF5 | IF6 | IF7 | IF8 | IF9 | IF10 | IF11 | IF12 |
| **Head height (nm)**  ***(hh)*** | Mean  SD  Median  Min  Max  25%  75%  MAD | 1.93  0.4  1.83  1.37  3.52  1.62  2.07  0.3 | 2.16  0.4  2.13  1.49  3.94  1.97  2.28  0.2 | 2.17  0.4  2.10  1.03  3.59  1.92  2.31  0.3 | 1.96  0.3  1.91  1.45  3.15  1.78  2.09  0.2 | 2.02  0.3  2.00  1.29  2.79  1.83  2.20  0.2 | 1.85  0.3  1.84  1.20  2.38  1.71  2.01  0.2 | 1.92  0.4  1.81  0.27  3.75  1.61  2.09  0.3 | 2.12  0.3  2.13  1.45  2.69  1.92  2.26  0.2 | 2.14  0.4  2.11  1.50  3.60  1.92  2.31  0.3 | 2.10  0.3  2.11  1.32  3.00  1.89  2.29  0.2 | 2.42  0.5  2.26  1.82  4.43  2.08  2.61  0.4 | 2.21  0.5  2.10  1.26  3.86  1.82  2.48  0.4 | 1.85  0.3  1.81  1,19  2.90  1.63  2.04  0.3 | 2.05  0.7  1.88  1.25  5.15  1.60  2.21  0.5 | 1.94  0.4  1.80  1.47  4.10  1.70  2.05  0.3 | 2.03  0.6  1.92  1.01  4.27  1.71  2.15  0.4 | 2.03  0.4  2.04  1.03  3.56  1.83  2.22  0.3 | 2.16  0.3  2.09  1.61  2.97  1.98  2.37  0.3 | 2.07  0.3  2.02  1.27  3.33  1.89  2.17  0.2 | 2.12  0.6  2.10  1.15  5.01  1.82  2.28  0.4 | 2.05  0.5  2.03  1.21  3.78  1.74  2.22  0.3 | 1.92  0.4  1.81  1.12  2.84  1.69  2.22  0.3 |
| **Head/Midpiece height (µm)**  ***(hmh)*** | Mean  SD  Median  Min  Max  25%  75%  MAD | 0.50  0.3  0.49  0.05  1.39  0.30  0.64  0.2 | 0.64  0.3  0.61  0.05  1.25  0.46  0.810.2 | 0.79  0.3  0.80  0.18  1.56  0.64  0.97  0.2 | 0.54  0.2  0.49  0.22  1.14  0.42  0.63  0.2 | 0.62  0.3  0.58  0.04  1.35  0.45  0.75  0.2 | 0.43  0.2  0.42  0.01  1.19  0.26  0.53  0.2 | 0.52  0.4  0.48  0.01  1.82  0.21  0.78  0.3 | 0.43  0.3  0.43  0.01  1.29  0.22  0.560.2 | 0.44  0.3  0.47  0.02  1.04  0.17  0.64  0.2 | 0.62  0.3  0.59  0.05  1.94  0.42  0.77  0.2 | 0.73  0.4  0.73  1.00  1.90  0.52  0.91  0.3 | 0.70  0.5  0.64  0.001  2.01  0.35  0.99  0.4 | 0.57  0.3  0.56  0.001  1.48  0.36  0.71  0.2 | 0.58  0.3  0.54  0.05  1.59  0.40  0.74  0.2 | 0.53  0.4  0.40  0.05  1.82  0.29  0.63  0.3 | 0.56  0.3  0.54  0.12  1.23  0.35  0.77  0.2 | 0.68  0.3  0.67  0.02  1.50  0.47  0.91  0.2 | 0.44  0.3  0.39  0.001  1.29  0.23  0.62  0.2 | 0.53  0.3  0.53  0.01  1.42  0.35  0.71  0.2 | 0.66  0.4  0.61  0.11  2.71  0.42  0.79  0.2 | 0.66  0.3  0.65  0.15  1.60  0.45  0.86  0.2 | 0.51  0.3  0.46  0.002  1.39  0.31  0.72  0.3 |
| **Acrosome/**  **nucleus height (µm)**  ***(anh)*** | Mean  SD  Median  Min  Max  25%  75%  MAD | 1.22  0.3  1.22  0.69  1.98  1.03  1.4  0.2 | 1.37  0.3  1.36  0.39  2.37  1.17  1.6  0.2 | 1.36  0.3  1.38  0.33  2.37  1.15  1.6  0.3 | 1.24  0.3  1.25  0.81  2.55  1.08  1.3  0.2 | 1.19  0.3  1.19  0.38  2.02  1.03  1.3  0.2 | 1.27  0.3  1.24  0.70  2.08  1.11  1.5  0.2 | 1.28  0.4  1.22  0.45  2.26  1.03  1.5  0.3 | 1.34  0.3  1.31  0.71  2.11  1.16  1.5  0.2 | 1.29  0.3  1.34  0.57  1.99  1.05  1.5  0.3 | 1.41  0.4  1.42  0.22  2.40  1.21  1.6  0.3 | 1.41  0.5  1.35  0.75  3.04  1.11  1.5  0.3 | 1.34  0.6  1.22  0.48  3.50  0.90  1.5  0.5 | 1.23  0.3  1.19  0.48  2.33  1.05  1.5  0.3 | 1.24  0.4  1.22  0.21  2.69  0.91  1.5  0.3 | 1.29  0.4  1.18  0.40  2.92  1.05  1.4  0.3 | 1.40  0.5  1.28  0.63  3.15  1.14  1.6  0.3 | 1.24  0.4  1.25  0.30  2.30  1.01  1.4  0.3 | 1.17  0.3  1.15  0.55  2.01  0.98  1.3  0.2 | 1.25  0.4  1.25  0.14  2.37  1.05  1.4  0.3 | 1.42  0.4  1.34  0.69  2.76  1.09  1.8  0.4 | 1.26  0.4  1.27  0.08  2.46  1.12  1.5  0.3 | 1.02  0.5  1.04  0.01  2.48  0.75  1.2  0.3 |
| **Volume loss (%)**  ***(vl)*** | Mean  SD  Median  Min  Max  25%  75%  MAD | 8.0  7.8  5  0  30  2.5  10  7 | 5.0  6.2  0  0  20  0  5  5 | 8.0  8.0  5  0  30  0  10  7 | 80  8.2  8  0  25  0  13  7 | 8.0  7.8  5  0  30  0  10  6 | 11.0  10.1  10  0  40  5  20  8 | 5.0  6.8  5  0  40  0  5  4 | 1.0  3.5  0  0  20  0  0  2 | 4.0  5.7  0  0  20  0  5  4 | 10  9.0  10  0  40  5  20  7 | 8.4  8.7  5  0  30  0  14  7 | 5.0  8.5  0  0  30  0  6  6 | 6.4  8.5  5  0  30  0  10  7 | 11.0  8.4  10  0  40  5  15  6 | 8.0  9.0  5  0  30  0  10  7 | 5.9  5.9  5  0  30  0  10  4 | 9.0  8.0  10  0  30  0  20  7 | 2.9  5.4  0  0  20  0  5  4 | 5.7  7.2  5  0  30  0  10  6 | 9.5  9.6  5  0  40  0  10  7 | 7.8  9.2  5  0  40  0  40  7 | 6.6  7.9  5  0  40  0  40  6 |

**Supplementary Table S6.** Descriptive statistics and comparison of spermatozoa three-dimensional parameters and volume loss obtained in fertile and infertile men by using DHM measurements of live sperm.

|  | **Head height (µm)**  ***(hh)*** | **Head/midpiece height (µm)**  ***(hmh)*** | **Acrosome/nucleus height (µm)**  ***(anh)*** | **Volume loss (%)**  ***(vl)*** |
| --- | --- | --- | --- | --- |
| **Fertile (n=10)** | | | | |
| Mean ± SD | 2.05 ± 0.4 | 0.57 ± 0.3 | 1.32 ± 0.3 | 0.07 ± 0.08 |
| Median | 2.04 | 0.53 | 1.29 | 0.05 |
| MAD | 0.3 | 0.2 | 0.3 | 0.07 |
| Min - max | 0.27 - 3.94 | 0.002 – 1.94 | 0.22 – 2.54 | 0 – 0.5 |
| 25% - 75% | 1.81 – 2.23 | 0.38 – 0.74 | 1.11 – 1.52 | 0 – 0.1 |
| skewness | 0.67 | 0.68 | 0.24 | 1.19 |
| kurtosis | 4.0 | 1.25 | 0.89 | 0.89 |
| **Infertile (n=12)** | | | | |
| Mean ± standard deviation | 2.07 ± 0.5 | 0.61 ± 0.3 | 1.27 ± 0.4 | 0.07 ± 0.08 |
| Median | 2.01 | 0.58 | 1.22 | 0.05 |
| MAD | 0.3 | 0.3 | 0.3 | 0.07 |
| Min - max | 1.01 – 5.15 | 0.10– 2.71 | 0.01 – 3.50 | 0 – 0.4 |
| 25% - 75% | 1.79 – 2.25 | 0.38 – 0.80 | 1.01– 1.47 | 0 -0.1 |
| skewness | 1.76 | 0.9 | 1.01 | 0.07 |
| kurtosis | 6.89 | 2.33 | 3.1 | 1.2 |

**Supplementary Table S7. S**tatistical differences in sperm parameters obtained by different methods of sperm separation using Percoll gradient and „swim-up” technique and DHM measurements of live sperm.

| Fertile indivi-  duals |  | Head length - hl | Head width - hw | Midpiece length - ml | Tail length – tl | **Head height – hh** | **Head/**  **midpiece height - hmh** | **Acrosome/**  **nucleus height -**  **anh** | **Density loss** | **Progressive motility** |
| --- | --- | --- | --- | --- | --- | --- | --- | --- | --- | --- |
| No 1 | S30/S60 |  |  |  | ***0.0198*** |  | ***0.0030*** |  |  |  |
|  | P47/P90 | ***0.0475*** |  |  |  |  | ***0.0536*** |  |  | ***0.0000*** |
|  | S60/P90 |  |  |  |  |  | ***0.0422*** |  |  | ***0.0000*** |
|  | S30/P90 |  |  |  |  |  |  |  |  |  |
|  | P47/S60 | ***0.0075*** | ***0.0985*** |  |  |  | ***0.0000*** |  |  | ***0.0050*** |
| No 2 | S30/S60 |  |  |  |  |  |  |  |  | ***0.0000*** |
|  | P47/P90 |  |  |  |  |  |  |  |  | ***0.0000*** |
|  | S60/P90 |  |  |  |  |  |  |  |  |  |
|  | S30/P90 |  |  |  |  |  |  |  |  | ***0.0000*** |
|  | P47/S60 |  |  |  |  |  |  |  |  | ***0.0000*** |
| No 3 | S30/S60 |  |  |  |  |  |  |  |  |  |
|  | P47/P90 |  |  |  | ***0.0002*** |  |  |  |  | ***0.0000*** |
|  | S60/P90 |  |  |  |  |  |  |  |  |  |
|  | S30/P90 |  |  |  |  |  |  |  |  |  |
|  | P47/S60 |  |  |  |  |  |  |  |  | ***0.000*** |
| No 4 | S30/S60 |  |  |  |  |  |  |  |  |  |
|  | P47/P90 |  |  |  |  |  | ***0.0000*** |  | ***0.0059*** |  |
|  | S60/P90 |  |  |  |  |  |  |  |  |  |
|  | S30/P90 |  |  |  |  |  |  |  | ***0.0274*** |  |
|  | P47/S60 |  |  |  |  |  |  |  |  |  |
| No 5 | S30/S60 |  |  | ***0.0208*** |  |  |  |  |  | ***0.0000*** |
|  | P47/P90 |  |  | ***0.0554*** |  |  | ***0.0097*** |  |  | ***0.0000*** |
|  | S60/P90 |  |  |  | ***0.0790*** |  |  |  |  |  |
|  | S30/P90 |  |  |  |  |  |  |  |  | ***0.0000*** |
|  | P47/S60 |  |  |  |  |  | ***0.0034*** |  |  | ***0.0000*** |
| No 6 | S30/S60 |  |  |  |  |  |  |  |  |  |
|  | P47/P90 |  |  |  | ***0.0430*** |  |  |  |  | ***0.0000*** |
|  | S60/P90 |  |  |  | ***0.0225*** |  |  |  |  |  |
|  | S30/P90 |  |  |  |  |  |  |  |  |  |
|  | P47/S60 |  |  |  |  | ***0.0187*** |  |  |  | ***0.0000*** |
| No 7 | S30/S60 |  |  |  |  |  |  |  |  |  |
|  | P47/P90 |  |  |  |  |  | ***0.0258*** |  |  |  |
|  | S60/P90 |  |  |  |  |  |  |  |  | ***0.0000*** |
|  | S30/P90 |  |  |  |  |  | ***0.0556*** |  |  | ***0.0000*** |
|  | P47/S60 |  |  |  |  |  |  |  |  | ***0.0000*** |
| No 8 | S30/S60 |  |  |  |  |  |  |  |  |  |
|  | S60/P90 |  |  |  |  |  |  |  |  |  |
|  | S30/P90 |  |  |  |  |  |  |  |  |  |
| No 9 | S30/S60 |  |  |  | ***0.0077*** |  |  |  |  |  |
|  | P47/P90 |  |  |  |  | ***0.0386*** | ***0.0000*** | ***0.0041*** |  | ***0.0005*** |
|  | S60/P90 |  |  | ***0.0191*** | ***0.0000*** | ***0.0000*** | ***0.0000*** | ***0.0003*** |  |  |
|  | S30/P90 |  |  |  |  | ***0.0030*** | ***0.0000*** | ***0.0011*** |  | ***0.0463*** |
|  | P47/S60 |  |  | ***0.0004*** | ***0.0000*** |  |  |  |  | ***0.0000*** |

**Supplementary Figure S1.** Distribution of two-dimensional sperm parameter values obtained by DHM measurements of fertile and infertile men samples; representative histograms. **A.** Visible sperm head length (µm) from fertile (F) and infertile (IF) men; 60 measurements. **Narrow concentration of obtained values for fertile men.** **B.** Visible sperm head width (µm) from fertile (F) and infertile (IF) men; 60 measurements. **Narrow concentration of obtained values for fertile men.** **C.** Visible sperm midpiece length (µm) from fertile (F) and infertile (IF) men; 60 measurements. Highly concentrated range of obtained values for fertile men. **D.** Visible sperm tail length (µm) from fertile (F) and infertile (IF) men; 60 measurements. **Narrow concentration of obtained values for fertile men.**


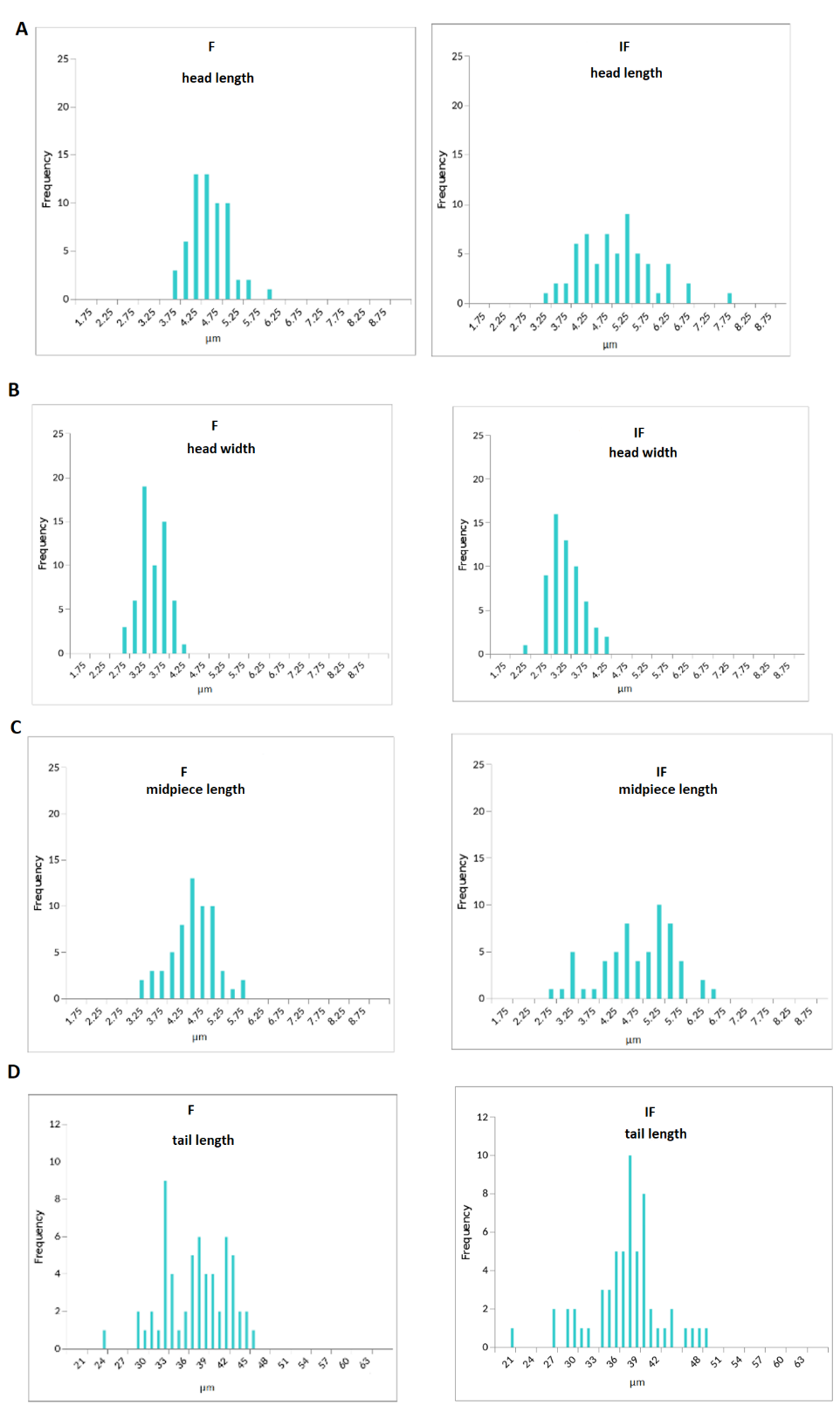


**Supplementary Figure S2.** *Head height* measurement. Screen dumps from the window showing a 2D-profile analysis obtained from DHM measurements of live sperm.


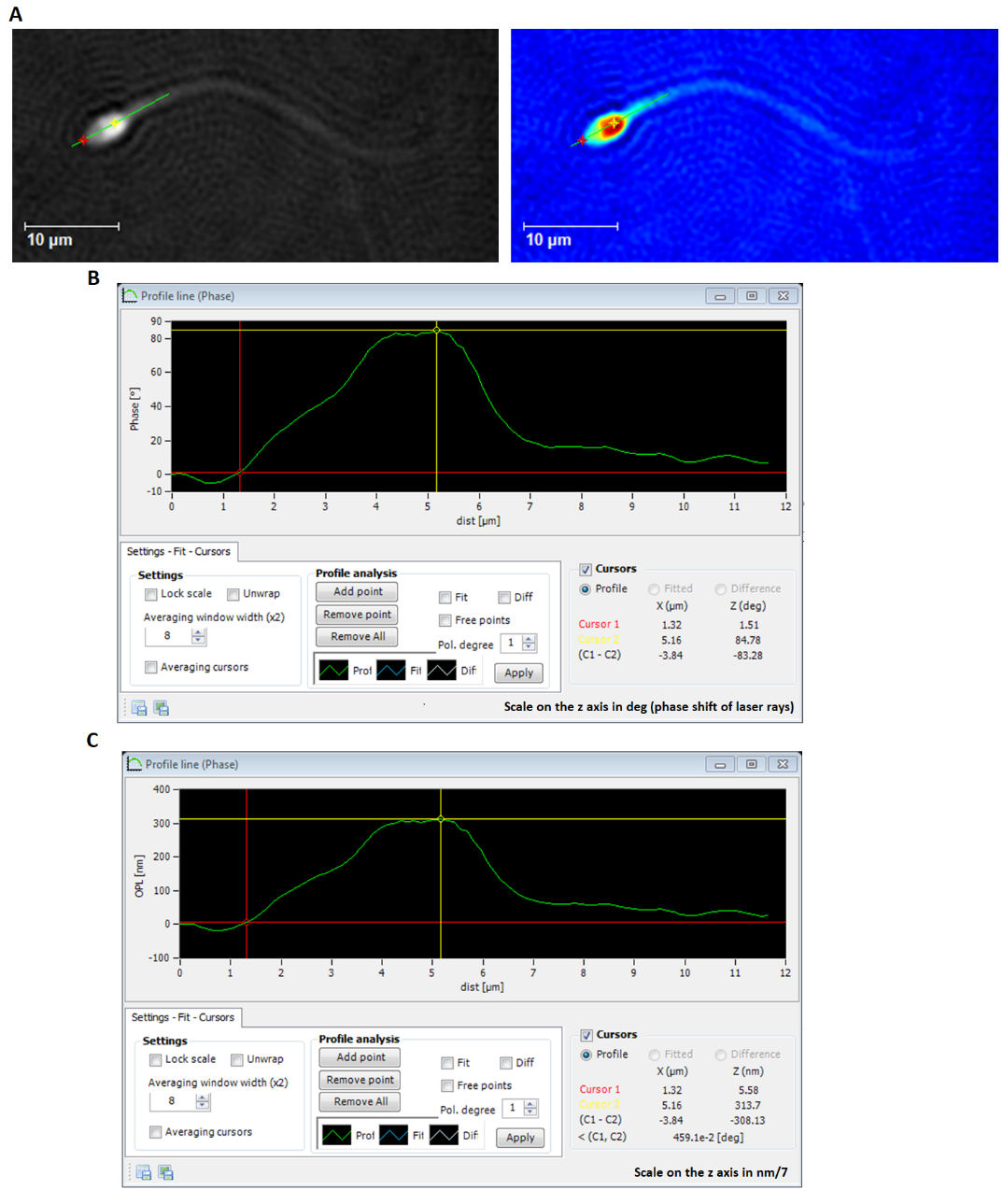


**Supplementary Figure S3.** *Head width* measurement. Screen dumps from the window showing a 2D-profile analysis obtained from DHM measurements of live sperm.


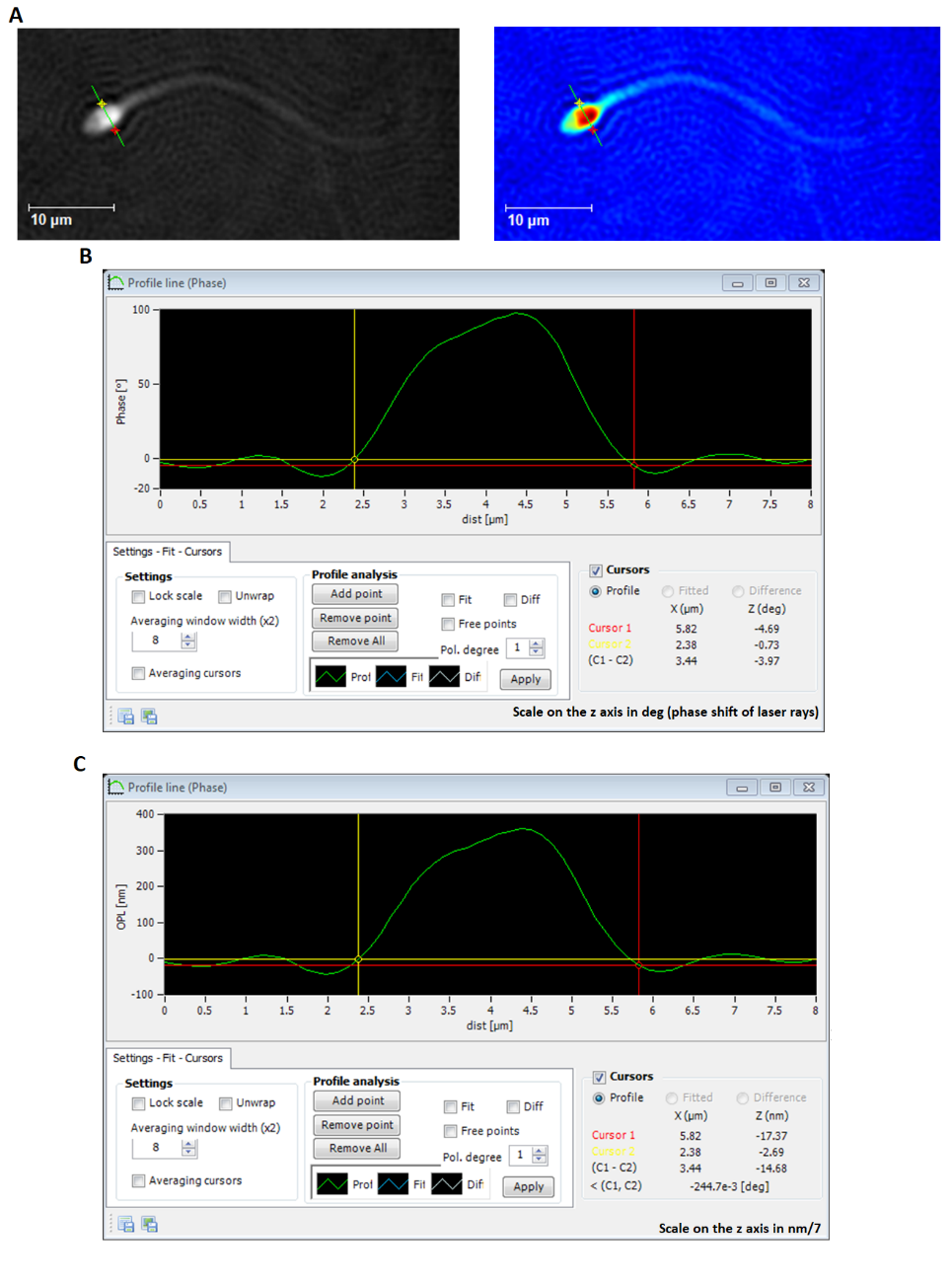


**Supplementary Figure S4. A**. *Head length*, **B.** *midpiece length*, **C**, *head width* measurement. Screen dumps from the window showing a 2D-profile analysis obtained from DHM measurements of live sperm.


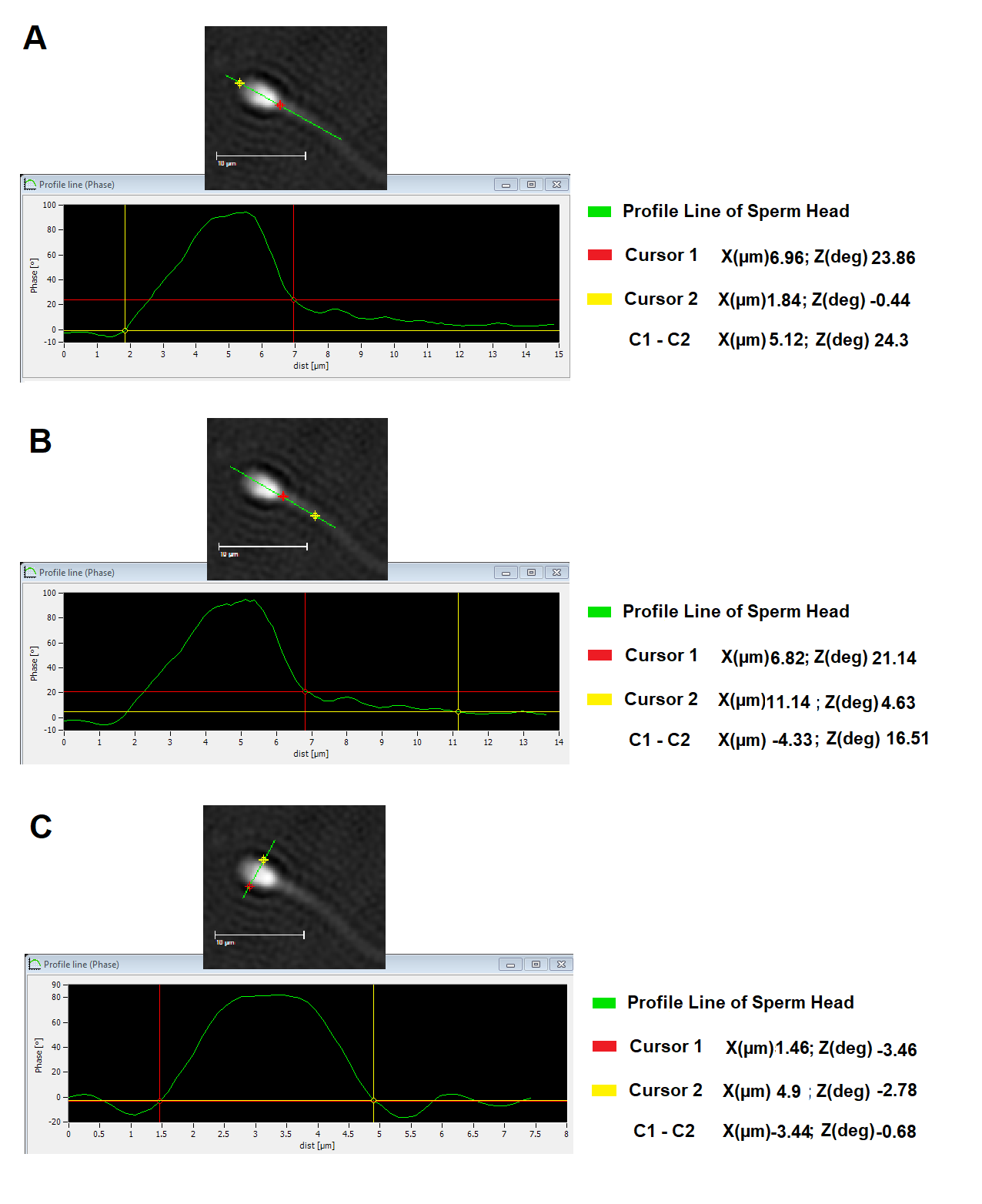


**Supplementary Figure S5.** *Head tail* measurement. Screen dumps from the window showing a 2D-profile analysis obtained from DHM measurements of live sperm.


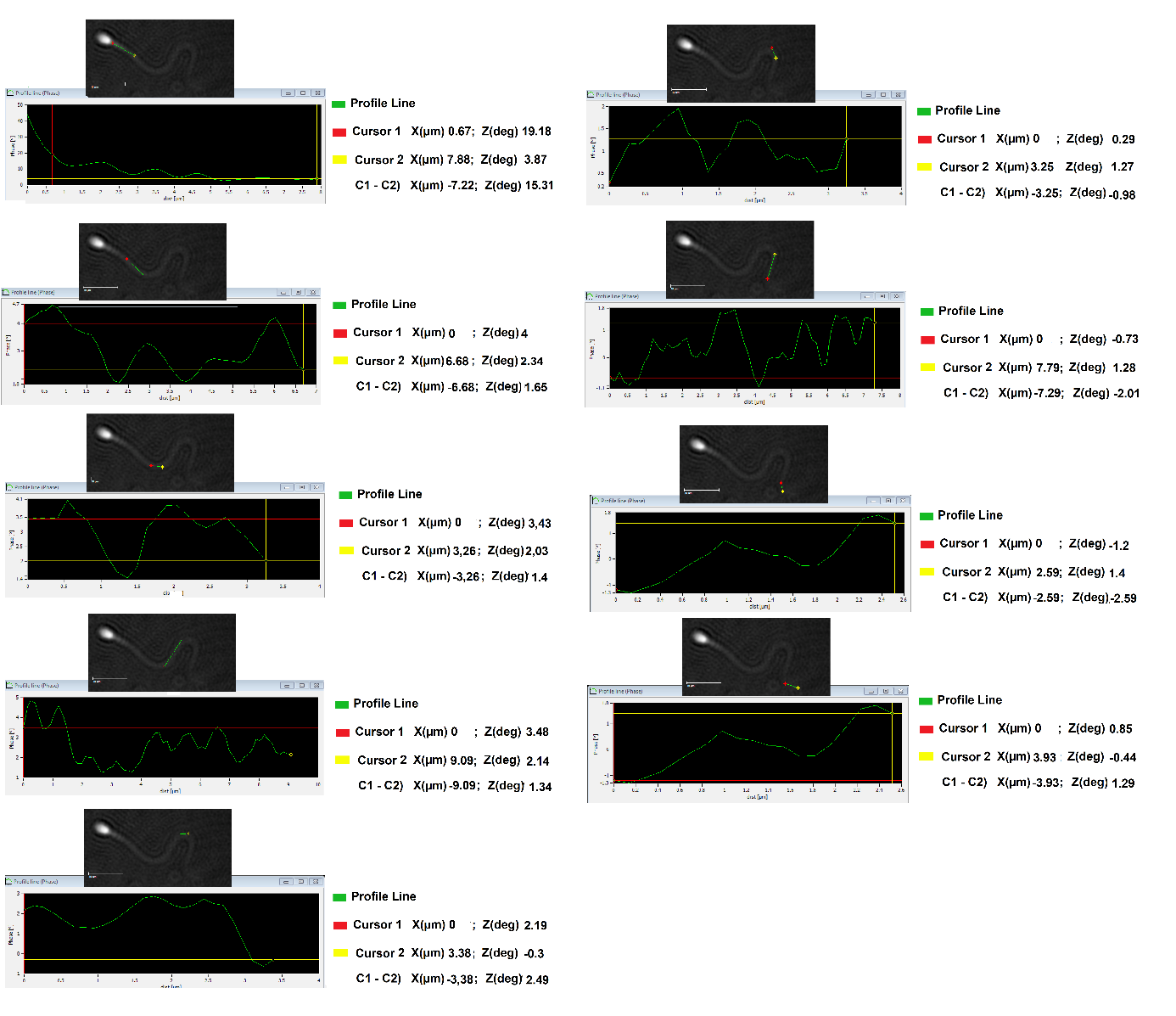


**Supplementary Figure S6. A**. *Acrosome/nucleus height,* **B.** *head***/***midpiece height* (cursor1), **C**. *head height* measurement. Screen dumps from the window showing a 3D-profile analysis obtained from DHM measurements of live sperm.


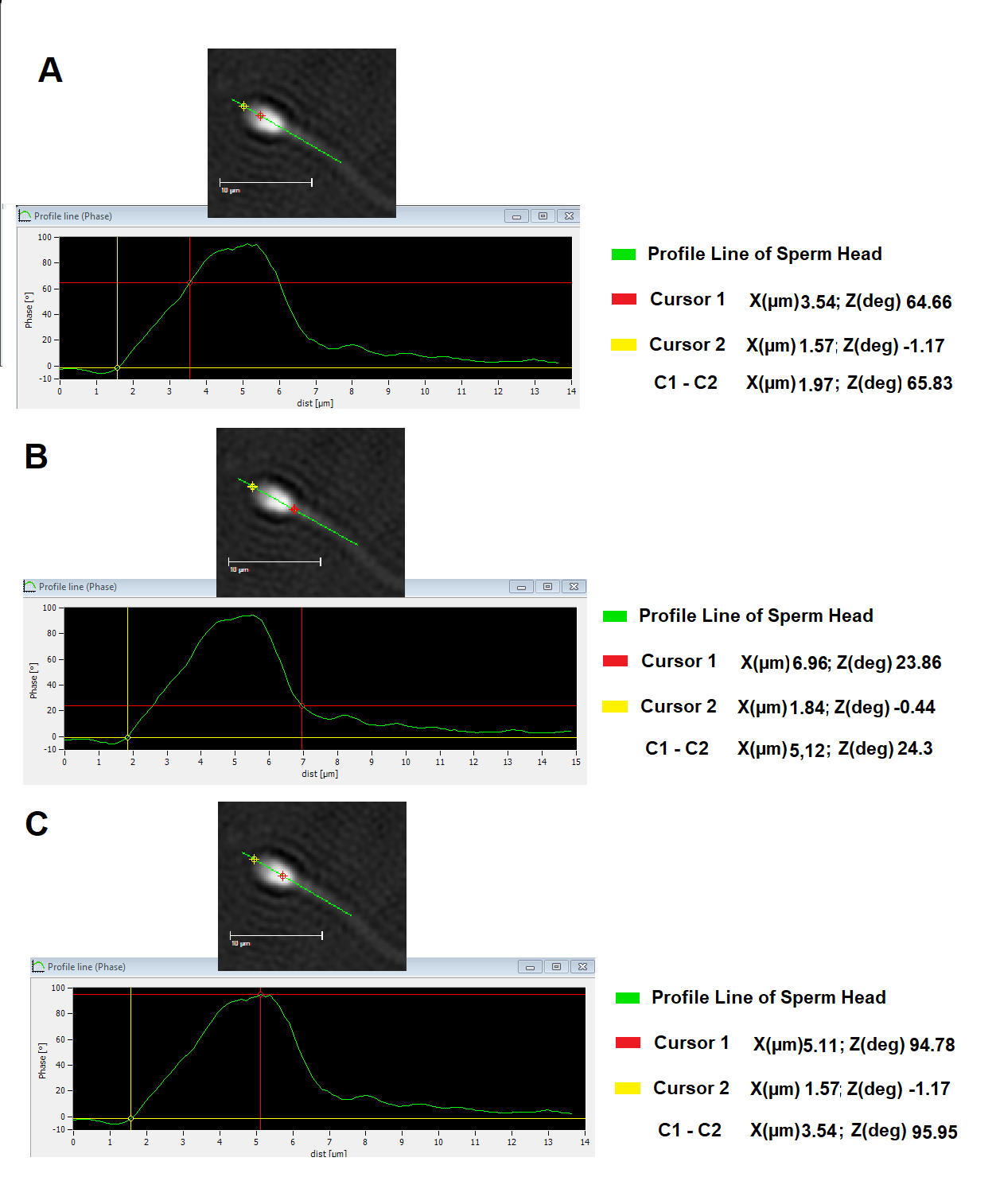


**Supplementary Figure S7.** *Vacuole* measurement. Screen dumps from the window showing a 3D-profile analysis obtained from DHM measurements of live sperm.


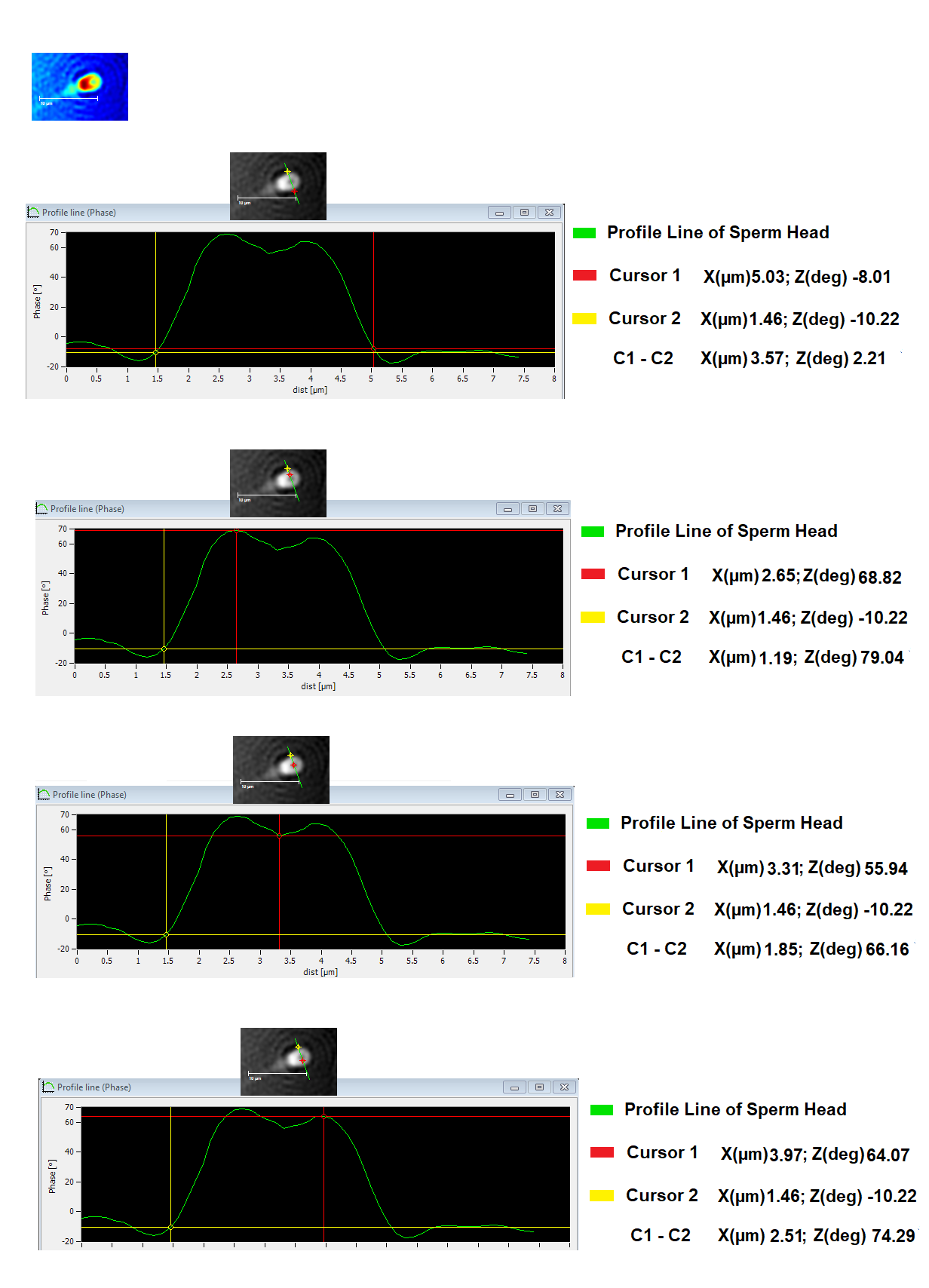


**Supplementary Figure S8.** *Density loss* measurement. Screen dumps from the window showing a 3D-profile analysis obtained from DHM measurements of live sperm.


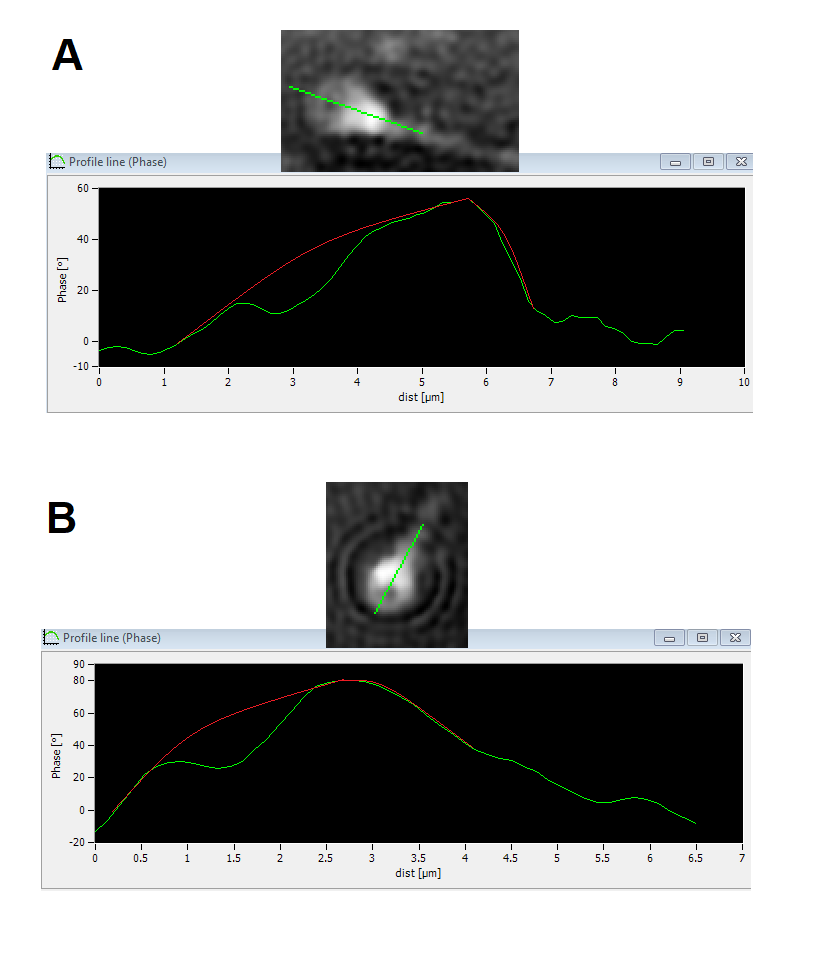


**Supplementary Figure S9.** Phase images and profiles of DHM measurements of the cells in the semen smear (a) and stained with Papanicolau method (b). Head width measurement results for the cells stained with Papanicolau method using the phase (b) and intensity image (c) ; *hw*=5.39 µm and *hw*=5.36 µm, respectively.

(a)


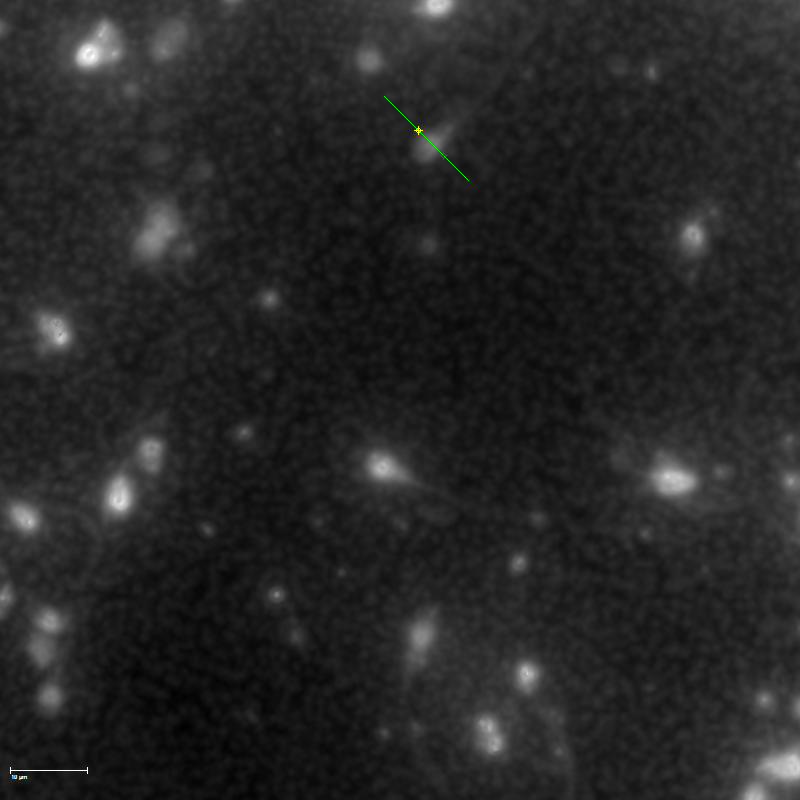

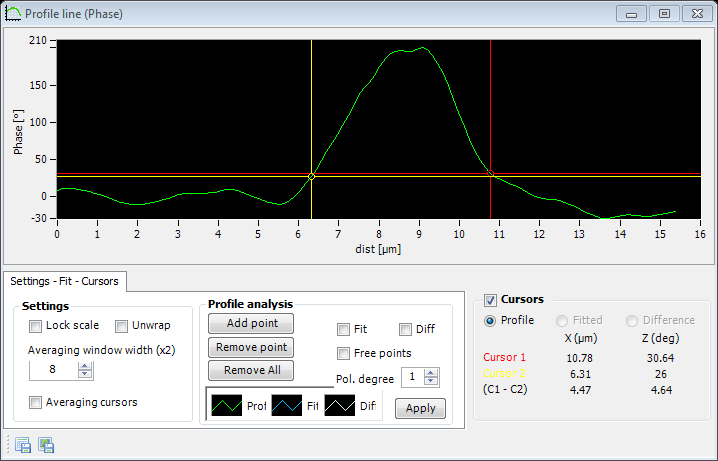


(b)


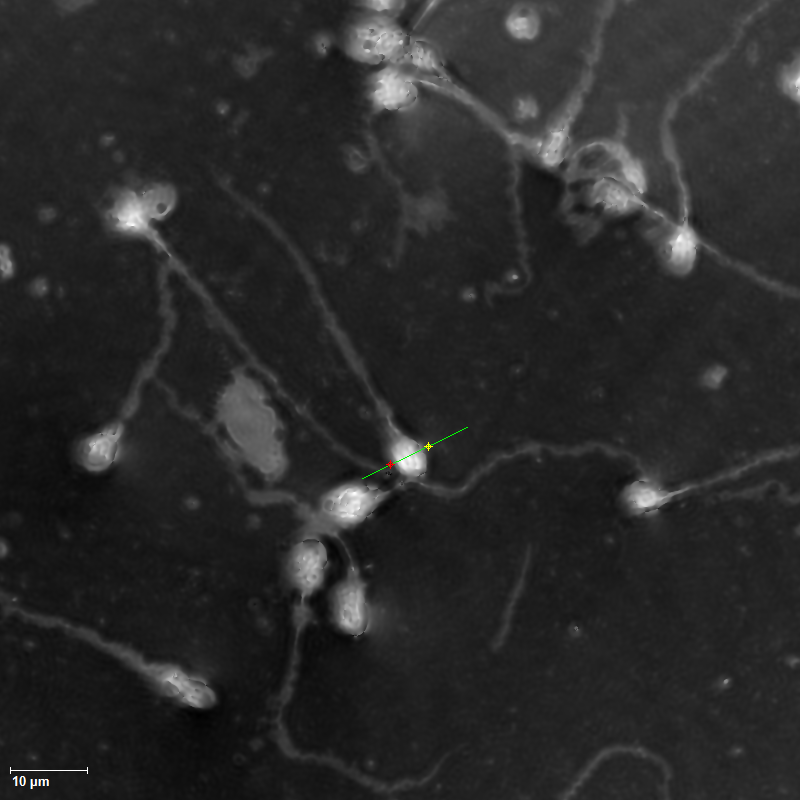

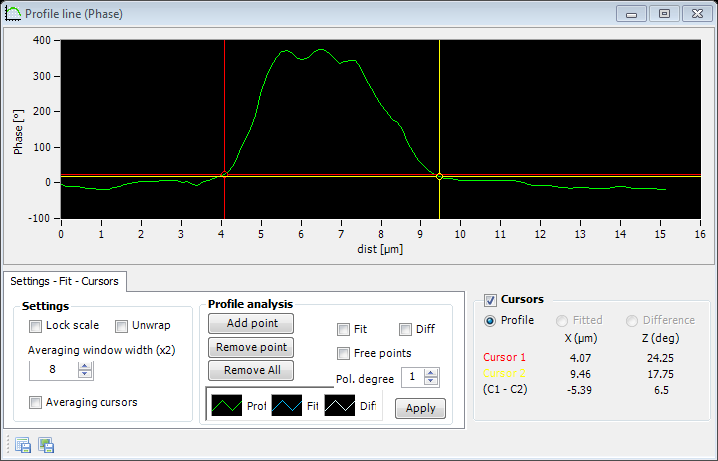


(c)


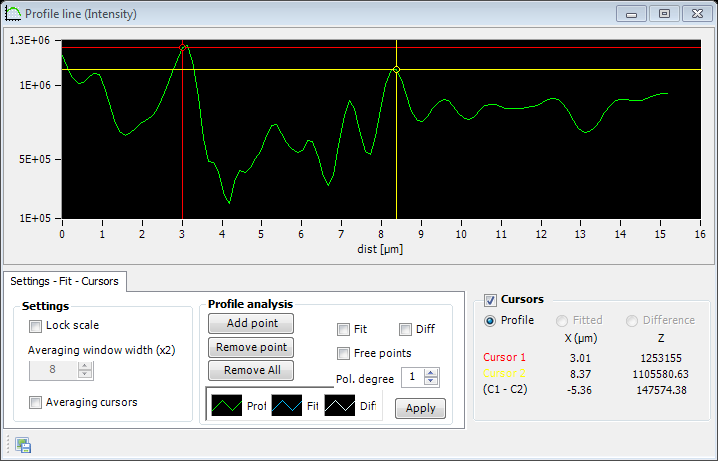

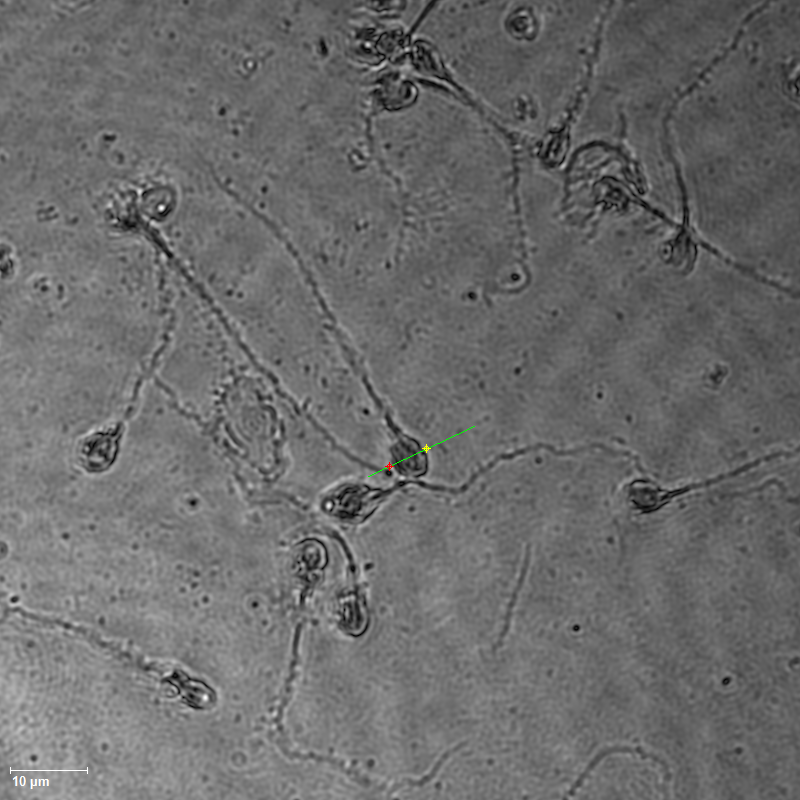


**Supplementary Figure S10.** Head width measurement results obtained by using DHM technique for the cells in liquid PBS using the intensity (a) and phase image (b) ; *hw* =2.39 µm and *hw* =2.39 µm, respectively.

(a)


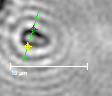

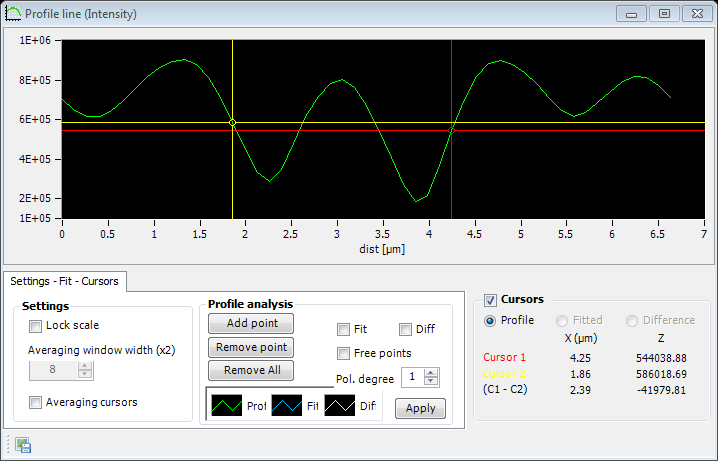


(b)


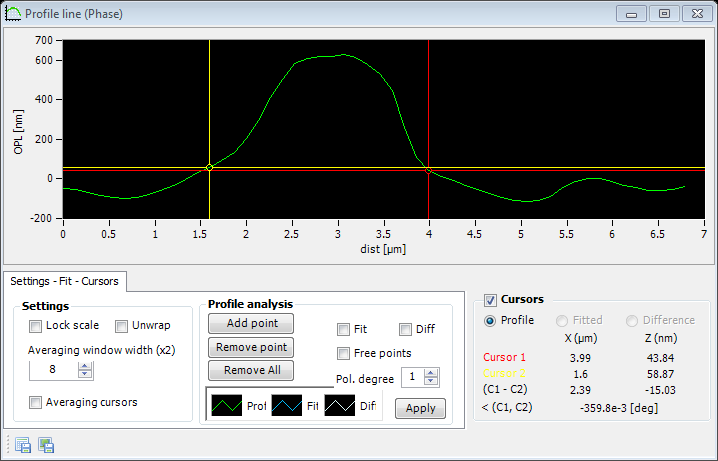

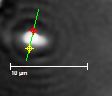


**Supplementary Figure S11.** Head width measurements results obtained by DHM technique for two different profile lines (a)-(b) and two different cursor positions (b)-(c); *hw* =3.6 µm and *hw* =3.57 µm and *hw* =3.83 µm, respectively.

(a)


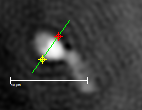

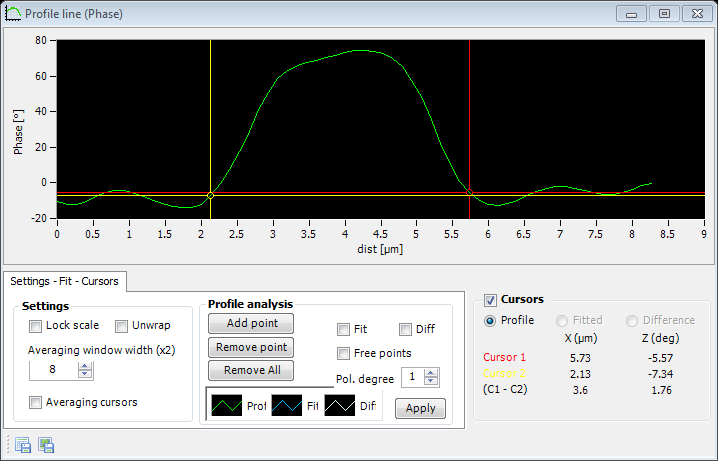


(b)


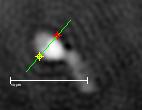

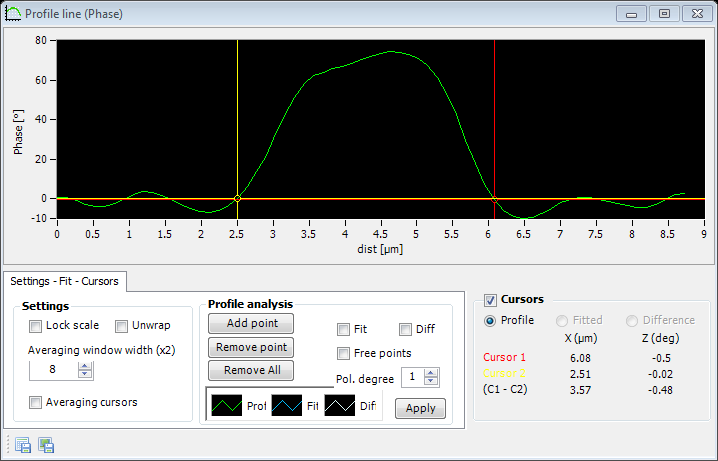


(c)


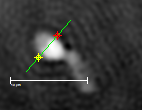

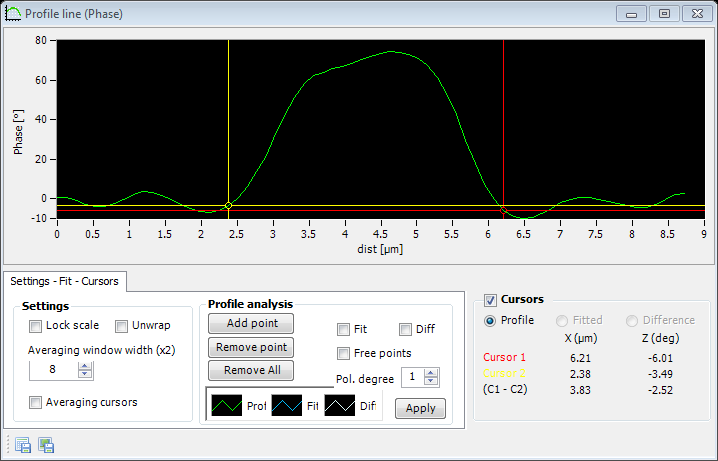


**Supplementary Figure S12.** Head height measurement results (in deg) for three digital adjustments of the focus distance; (a) f=-0.5 cm, (b) f=0.0 cm and (c) f=0.5 cm); *hh*=78,82 deg, *hh*=80,48 deg and *hh*=86.08 deg, respectively.

(a)


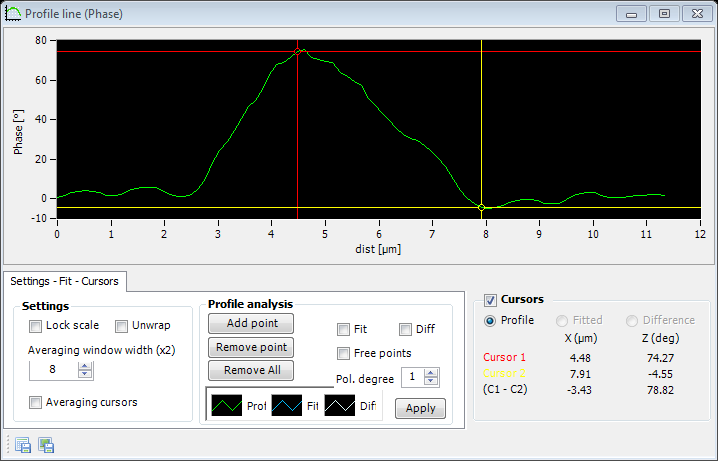

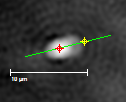


(b)


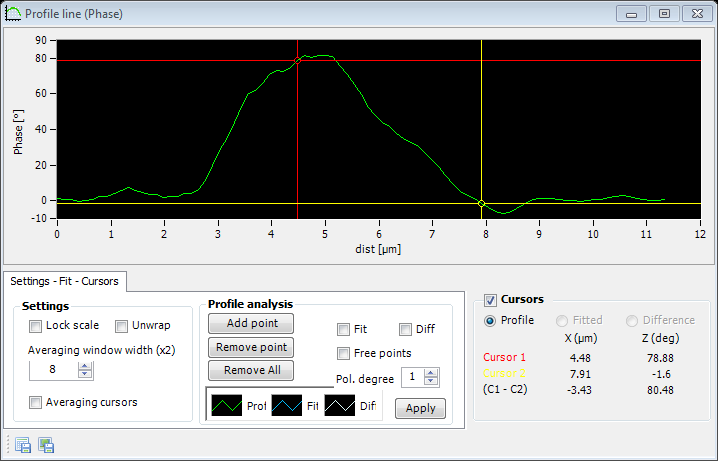

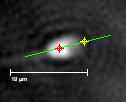


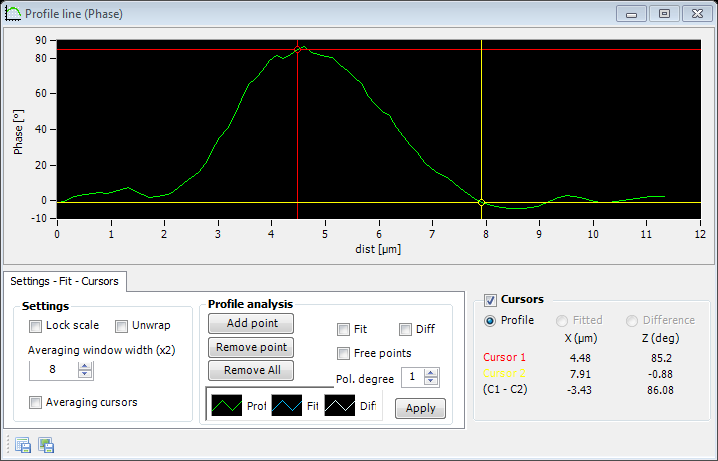
(c)


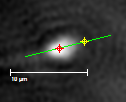

Supplement: Supplementary file 1 — Supplementary Information. [file 41598_2022_8798_MOESM1_ESM.docx]
